# Modeling nationalism, religiosity, and threat perception: During the COVID-19 pandemic

**DOI:** 10.1371/journal.pone.0281002

**Published:** 2023-04-06

**Authors:** Josh Bullock, Justin E. Lane, Igor Mikloušić, F. LeRon Shults

**Affiliations:** 1 Department of Criminology, Politics and Sociology, Kingston University, London, United Kingdom; 2 CulturePulse, Inc., Middletown, Delaware, United States of America; 3 ALAN Analytics, s.r.o, Slovak Academy of Science, Slovakia Lanova 2, Bratislava, Slovakia; 4 Institute of Social Sciences Ivo Pilar, Zagreb, Croatia; 5 Institute for Global Development and Planning, University of Agder, Kristiansand, Norway; Padjadjaran University: Universitas Padjadjaran, INDONESIA

## Abstract

The rise of nationalism and populism in Europe has created significant political and policy challenges. Understanding and addressing these challenges will require attention to the psychological mechanisms and social dynamics that have engendered and promoted these societal shifts. This article presents the results of two new empirical studies that attempt to shed light on the relationships between nationalism, religiosity, national and religious identification, threat perception, and sentiment toward different groups. Informed by identity fusion theory and moral foundations theory, Study 1 collected and analysed survey data on these topics. Study 2 utilized the results of Study 1 to construct a system dynamics model in which causal propositions and links are added to the variables, creating an artificial society within which hypotheses about these dynamics can be tested. Both the survey and the simulation suggest that nationalism and religion are affected by the same variables. As such, religion might not be a cause of nationalism (or nationalism the cause of religion), but they could be correlated because of mutual causation.

## Introduction

In recent decades, massive shifts have shaped Europe’s contemporary political landscape caused by geopolitical upheavals such as Brexit and COVID-19. How policymakers respond to the current pandemic could significantly affect how the world looks after the pandemic subsides. A region that has been defined by liberalism and freedom for the better part of a century has experienced a rise in nationalism and populism that has not been seen since after World War I. Historically, limitations on individual freedoms and new waves of legislation impeding freedom have accompanied the rise of nationalism and populism in Europe. These developments have also been accompanied by increased xenophobia and out-group distrust, which negatively affect economic cooperation globally and economic mobility locally. Therefore, understanding what motivates and drives populist and nationalist movements from a psychological and social perspective is critical to promoting policies that can promote individual freedom in Europe.

There are still significant gaps in the scholarly literature on populism and nationalism. Psychological studies often neglect the evolutionary basis for human psychological tendencies and motivations necessary for nationalist and populist movements. In particular, there is a lack of attention to the role of evolved human psychology in responding to persistent threats, which can fall into four broad categories in the literature: predation (threats to one’s life via being eaten or killed in some other way), contagion (threats to one’s life via physical infection), natural (threats to one’s life via natural disasters), and social (threats to one’s life by destroying social standing; exile from a group would have been equivalent to a death penalty in ancient ancestral environments) [1–3]. These threats have been discussed considering their effects on religion and other forms of behaviour, but they have not been employed to study nationalist and populist behaviours.

Since the start of the COVID-19 pandemic, multiple papers have begun to look at the relationship between COVID-19 and nationalism. on the assumption that due to recent events in the media, that nationalism is on the rise, particularly in reference to the political right-wing. For example, in a recent paper studying the impact of the pandemic on political trends, the authors state how their study revealed “that as non-conservatives (compared to conservatives) are more concerned with the virus, they are more likely to show an inclination of ideological validation. Given that their ideology advocates more tolerance, non-conservatives are less likely to support nationalistic policies” [4, p. 169]. This research has several shortcomings. For example, it utilized a CNN poll for its data and CNN is a news organization with well-established left-leaning political biases, which likely affected self-selection biases. The poll also deliberately over-selected participants in battleground states. In addition to sampling concerns, the research also may have conflated conservativism and nationalism in ways that are not valid as they used the support for the travel ban as a proxy for nationalism. This is problematic not only because valid measures of nationalism exist independently of any party or policy, but also because surveys from June 2020 showed that the majority of Americans across the political spectrum supported the travel ban and that the more research associated it with Trump, the less support was garnered for the travel ban [5]. Future research should opt to use direct measures of nationalism that can be replicated more generally, and which represent the cross-cultural phenomenon of nationalism, rather than base assumptions on a single policy support question.

A second study by Mula et al. [6] utilized much better methods and valid measures. However, its relevance to nationalism is only indirectly implied. The Mula et al. study focused on how cultural tightness can mediate the relationship between the COVID-19 threat and attitudes towards immigrants. This study was conducted using the PsyCorona dataset with over 63,000 participants from 115 countries. The survey included self-report measures of COVID-19 danger and an adaptation of the cultural tightness measure (validated previously in [7]) and directly asked participants about their attitudes towards immigrants. The survey was done over multiple waves to establish a baseline. Their results suggested that the COVID-19 threat was related to a greater desire for tightness, which was then linked to more negative attitudes towards immigrants. This study, however, did not directly address the issue of nationalism more generally or how national identities and political beliefs might be affected by COVID-19. As such, we believe that a more holistic explanation can be garnered by looking at the role of multiple threats in affecting political beliefs, which interact in complex ways with social identities to promote nationalism and/or exclusion against immigrants.

In what follows, two studies are presented that begin to fill this gap in the literature. The first is a survey used to inform our theoretical framework and explore possible relationships in an online sample. The second is a computer simulation study. Both studies described below (completed in 2020) found very clear effects among the relevant variables, enabling us to identify trends that require further explanation and calling for additional research as we move toward models capable of adequately informing policy discussions.

### Theoretical background

Although many theories from a variety of disciplines bear on the relationships between threat and populism/nationalism studied in this paper, two sets of sometimes overlapping literatures are essential for our purposes here. The first is *identity fusion theory* [8–12], which is closely linked to the concept of “sacred values.” Gómez [13] points out that the current challenge of the COVID-19 pandemic is converting some citizens into “devoted actors” and increasing fusion with various groups. When contagion threats strengthen nationalism, this intensified sense of unity may strengthen an in-group, but it also poses many potential problems for out-group dynamics and intergroup conflict. Gómez argues these changes may include “denial of the group’s wrongdoings… willingness to participate in extreme forms of protest on behalf of the group; maximizing the ingroup’s advantage over the outgroup even at one’s personal expense; protecting the group’s reputation… relative intergroup formidability… and the desire to retaliate against outgroup members” (p.2-3). All of this contributes to out-group hostility. In the US context, culpability has sometimes been consigned to the Chinese, with major media outlets and government officials, including the president, repeatedly using the term “Chinese virus,” which further entrenches the notion that the pandemic is the fault of “others.” This perpetuates the idea that “foreigners are also associated with semantic concepts that connote disease” [14, p. 333].

The second body of literature relevant for our current purposes is the literature on *moral foundations theory* which posits that human morality evolved with five distinct “foundations,” which are differentially distributed within human populations. Combining these five emotionally charged moral foundations forms the basis of each individual’s normative preferences: care/harm, fairness/cheating, loyalty/betrayal, authority/subversion, and purity/degradation. The first two foundations are sometimes referred to as “individualizing,” while the latter three are considered “binding.” Liberals tend to be guided primarily by the first two of these dyads, suppressing the other three, while conservatives rely on all five when making moral judgments [15–17]. Both of our studies were motivated by our interest in discovering the causal interactions among threats (of the four types mentioned above), religiosity, nationalism, and intergroup conflict. However, our evolutionary framework focuses on the role of threat perception, rather than moral belief commitments, as a factor that differentiates the poles in the political spectrum.

## Study 1

Study 1 collected data on the relationships between nationalism, religiosity, national and religious identification, threat perception, and sentiment toward different groups (using the same measures as the World Values Survey), particularly focusing on immigrants. Data was also collected on social media use and consumption of TV-based media. Participants were also asked about their experience during the Covid-19 pandemic and their infection status.

### Methods

An online survey was developed and deployed online with Alchemer (previously SurveyGizmo). Participants (N = 70) were recruited online by posting on forums and sharing on online social networks. Most participants were recruited on MTurk (N = 501). The first information presented to the participant was informed consent. Informed consent was provided by all participants, at the informed consent prompt, the participant was informed about the use and anonymization of the data and that survey responses guarantee the anonymity of each participant. All participants were aged 18 and over.

The data were analysed using several descriptive and statistical methods to explore the relationships between nationalism, threats, and other factors, such as nationality and gender. To test for differences between countries, we utilized an ANOVA, while for predicting nationalism from threat, we utilized linear regressions, or GLM depending on the data types of the independent variables. These analyses helped elucidate the complex relationships in the data and test key hypotheses. Lastly, we also utilized structural equation modelling, to assess the extent to which more complex and potentially causal models could be tested for their plausibility. Lastly, we were specifically interested in what would cause individuals to take a stance of not welcoming immigrants. Holding such a perspective could suggest that nationalist sentiment—which may function positively when it comes to motivation to support a government, pay taxes, or volunteer for civil or military service—has co-opted negative features of group-tribalism that could not only hinder larger-scale cooperation in the multi-ethnic contexts of the global Liberal socio-economic system, but could also result in inter-personal harm between individuals of different backgrounds. As such, we utilized machine learning techniques to investigate what features were most predictive of holding unwelcoming attitudes towards immigrants. This method involved utilizing a machine learning algorithm which was run over our data set several times, looking to fit a model where the most important features that are indicative of answering that they would not like to have immigrants as neighbours (the same question used to assess immigrant sentiment in the World Values Survey). In combination, all these methods are aimed at trying to interpolate what the key causal factors are in nationalism and how it may be affected by both culture and context of high-threat scenarios such as the socio-political and economic turmoil of the COVID-19 pandemic.

#### Ethics

All participants and their data are assumed to have the widest data rights in accordance with The Data Protection Act 2018 (UK); GDRP rights are also extended to non-UK citizens regardless of location or residency and data can be deleted (right to be forgotten) at the request of a participant at any time. All research was limited to anonymous survey information with no additional personal information recorded or analyzed beyond that shown in the survey to the participants. All research was performed in accordance with the Declaration of Helsinki. This research was approved by the internal ethical review board at ALAN Analytics s.r.o., in agreement with the University of Kingston. All research was performed in accordance with the relevant guidelines and regulations and data is made open source for future use in the electronic appendix.

#### Measures

The key variables were assessed using the following diverse set of measures:

*Covid-19 infection status*. Participants were asked if they had tested negative for Covid-19, if they had symptoms and weren’t tested, if they currently had Covid-19, or if they had recovered from Covid-19.

*Nationalism*. We used an adapted version of the Nationalism Scale first put forward by Mansillo [18]. The key addition was an item with the prompt “to be truly part of my nation, one must be a specific race or ethnicity”. We found the original scale items to have acceptable reliability (α = .88; 95%CI = [.87;.89]), as well as the extended scale with our additional item (α = .90; 95%CI = [.89;.90]).

*Social Identification*. Participants were asked to state their nationality. They were then prompted to complete the Postmes 4-item social identification scale where the target group was their nation [19]. We found the scale items to have acceptable reliability (α = .92; 95%CI = [.91;.93]). Participants were further asked to state their religious affiliation. They were then prompted to complete the Postmes 4-item social identification scale where the target group was their religious group. We found the scale items to have acceptable reliability (α = .94; 95%CI = [.93;.95]).

*Fusion*. Individuals were also asked to state their nationality. They were then prompted to complete the verbal fusion scale where the target group was their nation [20]. We found the scale items to have acceptable reliability (α = .95; 95%CI = [.945;.953]). Participants were also asked to state their religious affiliation. They were then prompted to complete the verbal fusion scale where the target group was their religious group. We found the scale items to have acceptable reliability (α = .96; 95%CI = [.95;.96]).

*Supernatural Belief*. To measure supernatural beliefs, an adapted version of the Supernatural Belief Scale [21] was used. The amendment involved the inclusion of two additional items. The first presented the prompt “There exists a universal force of justice that you could call karma”. The second was that “The universe has the ability to affect the events in our lives and provide balance.” We found the original scale items to have acceptable reliability (α = .98; 95%CI = [.975;.978]), as well as the extended scale with our additional item (α = .98; 95%CI = [.975;.978]).

*Threat*. The threat was assessed with a scale that we devised for the purpose of this study to measure four key evolutionary threats (predation, contagion, social, and natural hazards). We added the additional dimension of financial threats because of the likelihood that participants’ key concern during the Covid-19 pandemic could be the loss of their job. All items were based on a 5-item Likert scale (strongly disagree to strongly agree).

*Personality*. We used a standard 10-item Big-5 measure. We tested whether there was a significant fit for the 5-factor model and did find a significant fit (.78) using varimax rotation. The resulting root mean square of the residuals was found to be 0.09 (chi-squared = 1516.21 p < .01) justifying the use of the five-factor measure as intended.

*Religious attendance*. Participants were asked how frequently they attend religious services using a 10-item ordinal scale from daily to never.

*Social media use*. Participants were asked how much time they spend on social media (from rarely, to more than 2 hours a day).

*Brexit*. Participants from the UK were asked how they voted in the 2016 EU referendum and if they still support that decision.

*Politics*. Participants were first asked to report how liberal or conservative they are on economic issues using a visual slider anchored at very liberal and very conservative.

They were then asked to report how liberal or conservative they are on social issues using a visual slider anchored at very liberal and very conservative.

Lastly, they were asked what party they voted for in the last election, if any.

*Outgroups*. Using a Likert scale from very negative to very positive, participants were prompted to answer how they feel toward the following groups: Jews, Immigrants, Atheists, people of other races than their own, Christians, and Muslims. We also used questions taken from the World Values Survey, which prompted the participant with, “On this list are various groups of people. Could you please mark any that you would not like to have as neighbours?” Participants are then presented with the options: “drug addicts; people of a different race; people who have aids; immigrants/foreign workers; homosexuals; people of a different religion; heavy drinkers; unmarried couples living together; people who speak a different language”.

*Media use*. Participants were asked how frequently they watch or read the news online (from less than once a day to 3+ times a day)

*News Sources*. Participants were also asked to list the news sources they follow.

*Participants*. After removing invalid responses, we were left with N = 2,018 participants. Invalid responses were deemed to be those that were too incomplete for use or did not follow directions. The sample had 1038 men, 970 women, and ten other. Participants had a mean age of 39.06 (*sd* = 13.00).

### Results

#### Religious demographics

**Fig 1:**
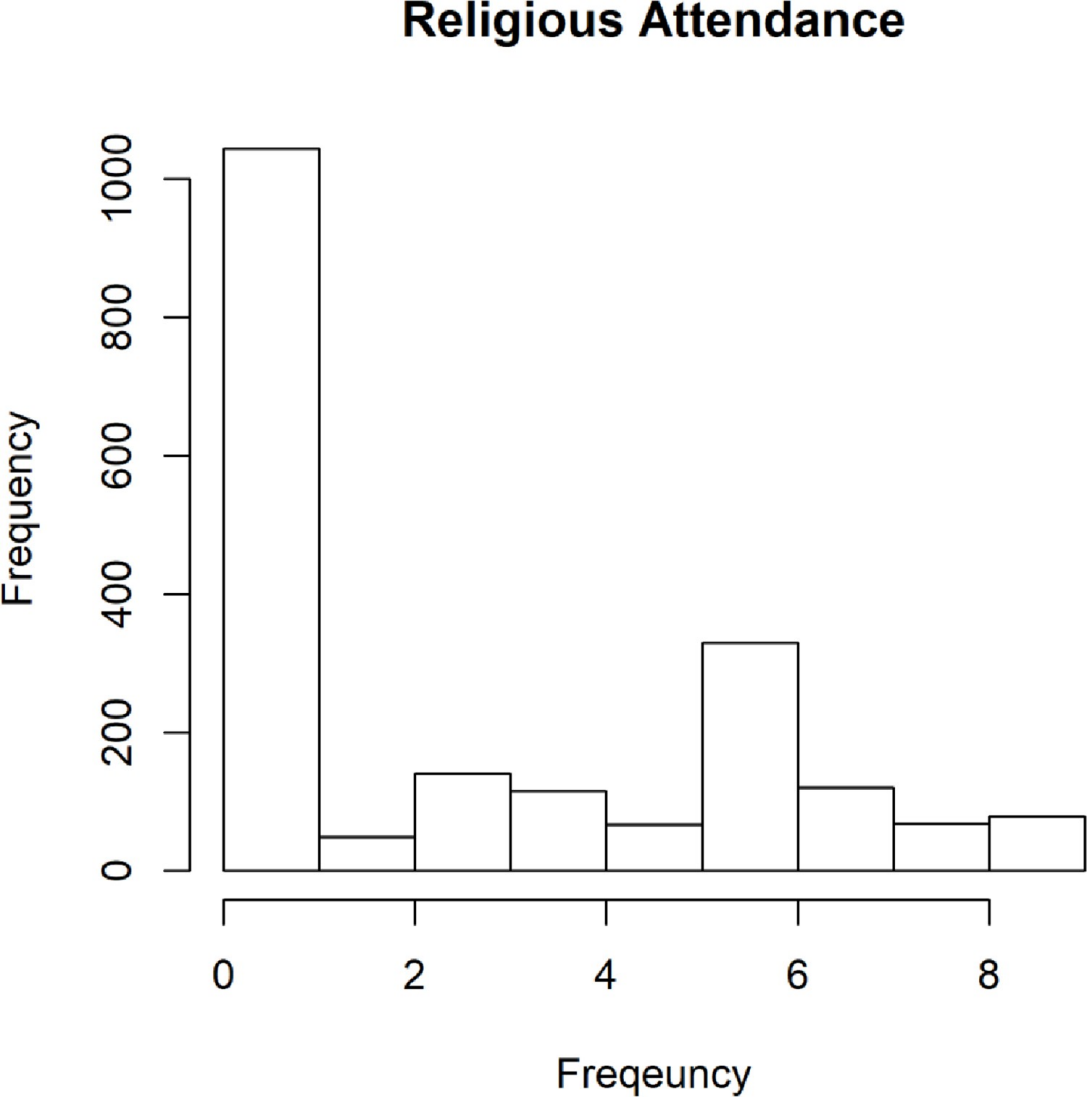
Distribution of frequency of religious attendance.

Generally, we found the sample to include a disproportionately large segment of participants reporting that they never attend religious services (distribution shown in Fig 1).

This distribution is consistent with the categorical self-identification responses (shown in Table 1).

**Table 1:** Religious identities of participants.

Religion	Frequency
Agnostic	169
Atheist	485
Buddhist	33
Catholic	481
Church of England/Anglican	37
Evangelical/Pentecostal/Charismatic	40
Hindu	64
Humanist	70
Jewish	26
Muslim	35
None	174
Other	39
Protestant (misc.)	246
Spiritual but not religious	119

#### Religious identity and beliefs

**Fig 2:**
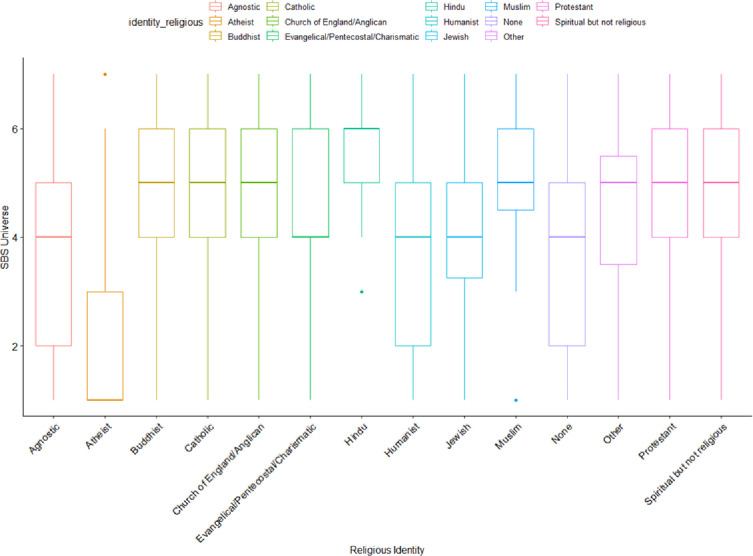
Strength of supernatural beliefs by religious identity.

Responses on the supernatural belief scale, however, suggested that supernatural belief was still present in the majority of the population (detailed below in Fig 2).

In addition to the standard supernatural belief scale, we added two items for karma and universal force (described in the measures section). The variation of support for those two beliefs revealed that these beliefs are held by individuals who are otherwise “nonreligious”.

Regarding the idea of a “universal force” there was general acceptance however significant differences were found between groups (*F*_(13,2000)_ = 89.47, *p* < .01)). As shown in Fig 2, Atheists had a long-tail distribution ranging from no support to a tailing off support, but we see somewhat normal distributions in this belief for Agnostics, Humanists, and nones. The Spiritual but not Religious have a similar signature to Protestants, trending toward acceptance of the idea.

**Fig 3:**
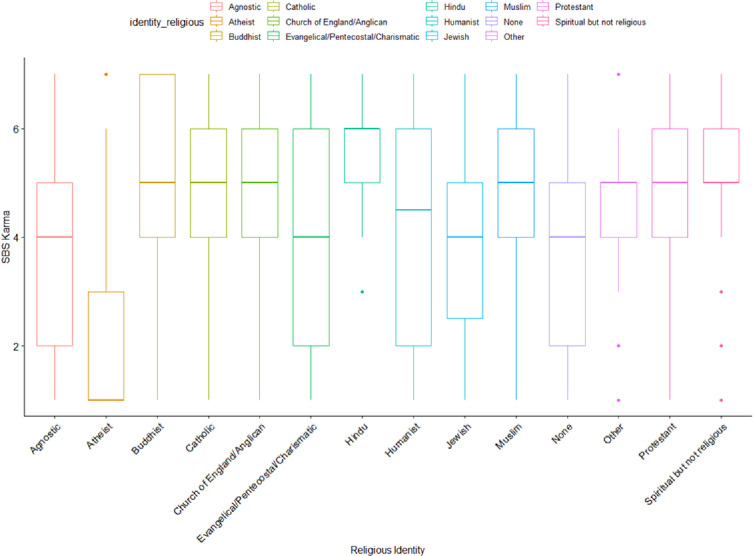
Belief in karma by religious identity.

In Fig 3, a similar pattern can be seen for the idea of karma, which appears to be more universally acceptable among all groups. However, we did find significant differences in the strength of support between religious groups (*F*_*(13*,*2000)*_ = 80.55, *p* < .01)). This is particularly interesting as Karma is the hallmark of eastern religions, but it nevertheless appears acceptable to many western nonreligious individuals. The distribution of belief in karma is presented in the figure below.

#### Political identity and beliefs

**Fig 4:**
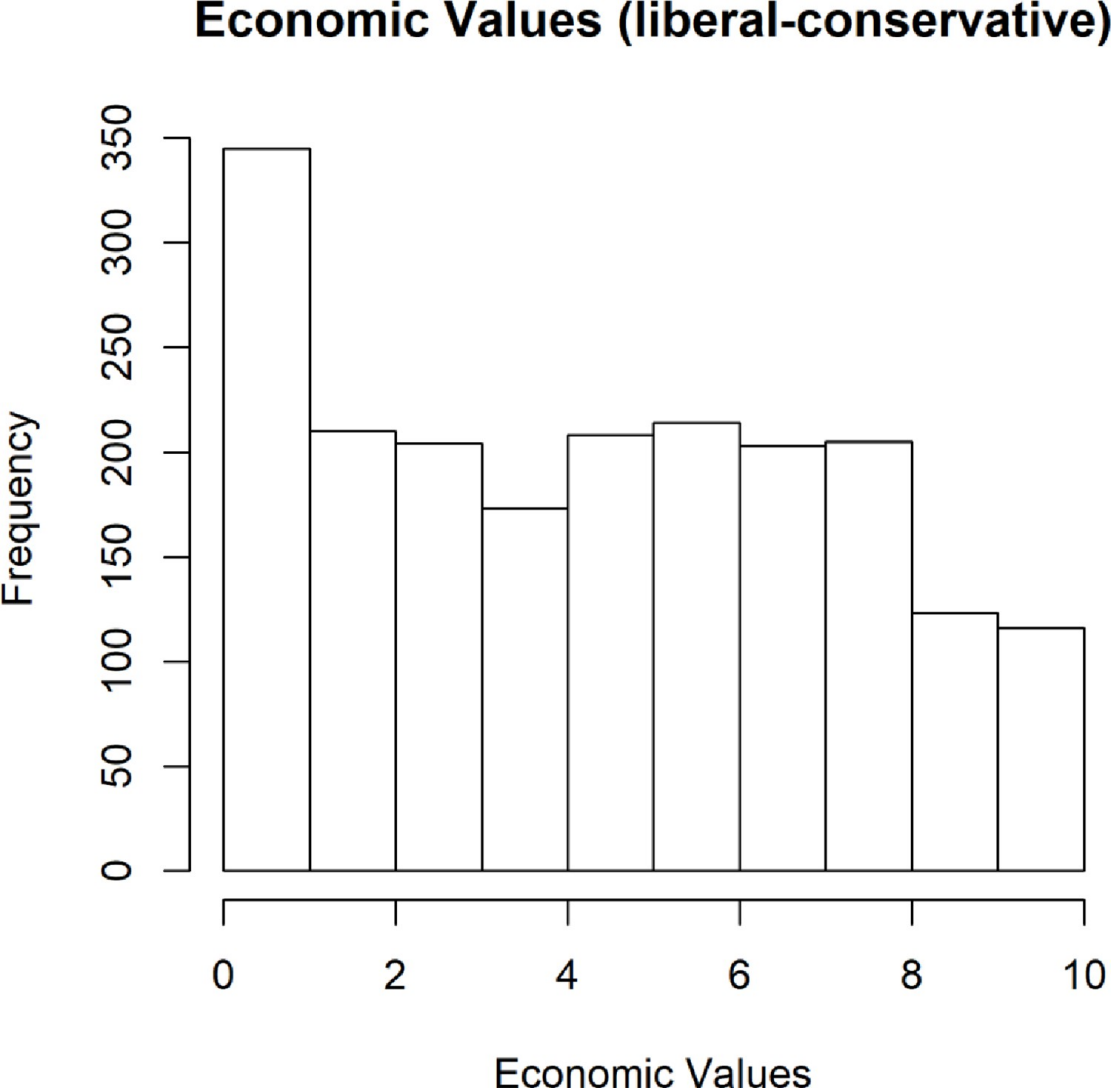
Participants by self-reported economic values (0-most liberal; 10-most conservative).

We found that our sample had a nearly uniform distribution of economic values (Fig 4), with there being a slight overrepresentation of people stating that they are “very liberal” in economic values and a lower number of those stating that they are “very conservative”.

**Fig 5:**
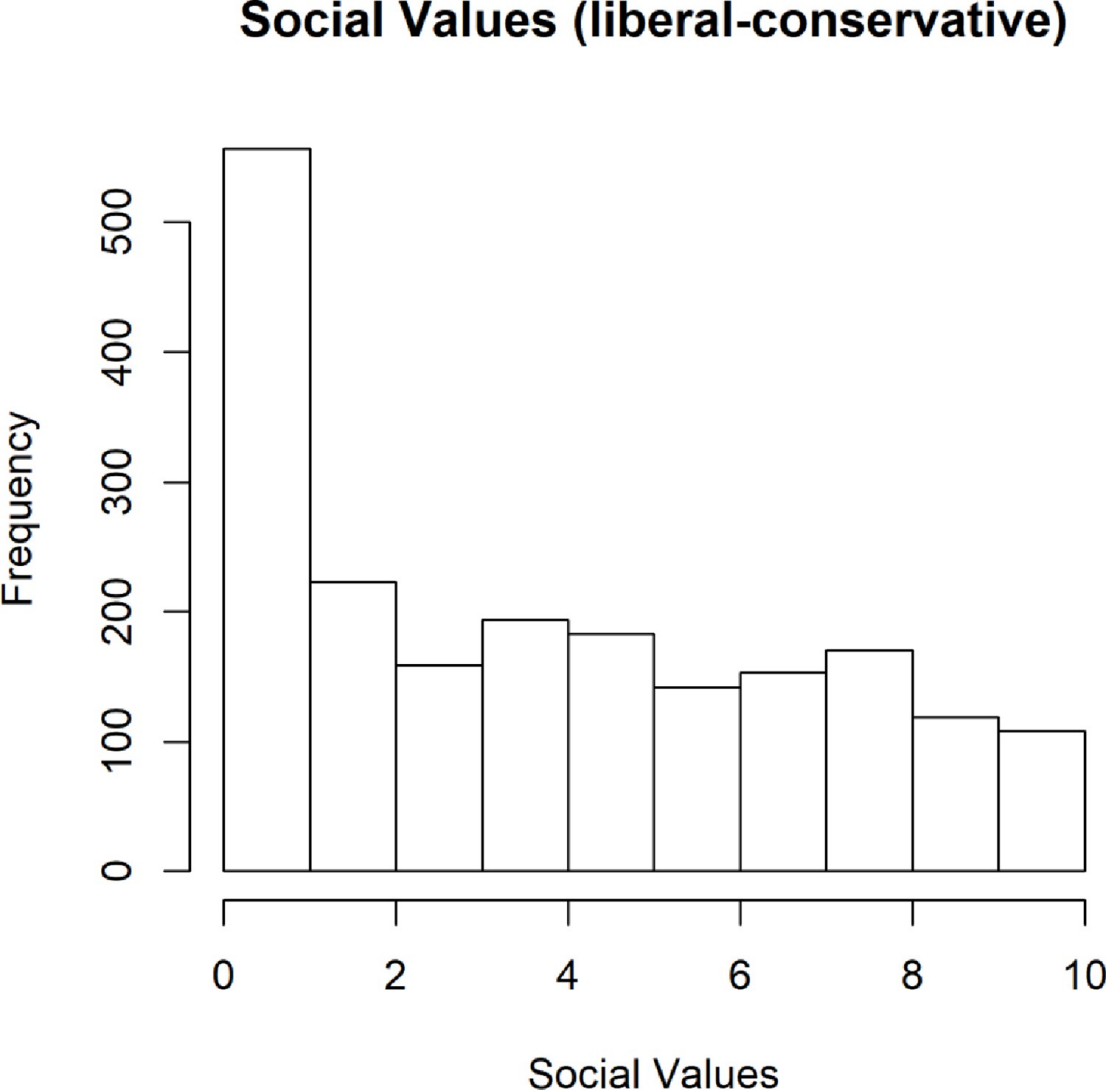
Distribution of social political values (0- most liberal; 10-most conservative).

This over-representation of very liberal participants was exacerbated when it comes to the participants’ self-reports on social values, where the distribution began to resemble a long tail distribution weighted toward very liberal social values (Fig 5). While this may not appear consistent with the general population, it is consistent with many online communities [22]. To date, we are not aware of any investigation of the MTurk population in this regard; however, it is telling that it was overweighted in our sample. We do not believe that recruiting participants from social networks, which are well-known to lean more liberal in political persuasion, can account for this overall distribution.

#### Nationalism

**Fig 6:**
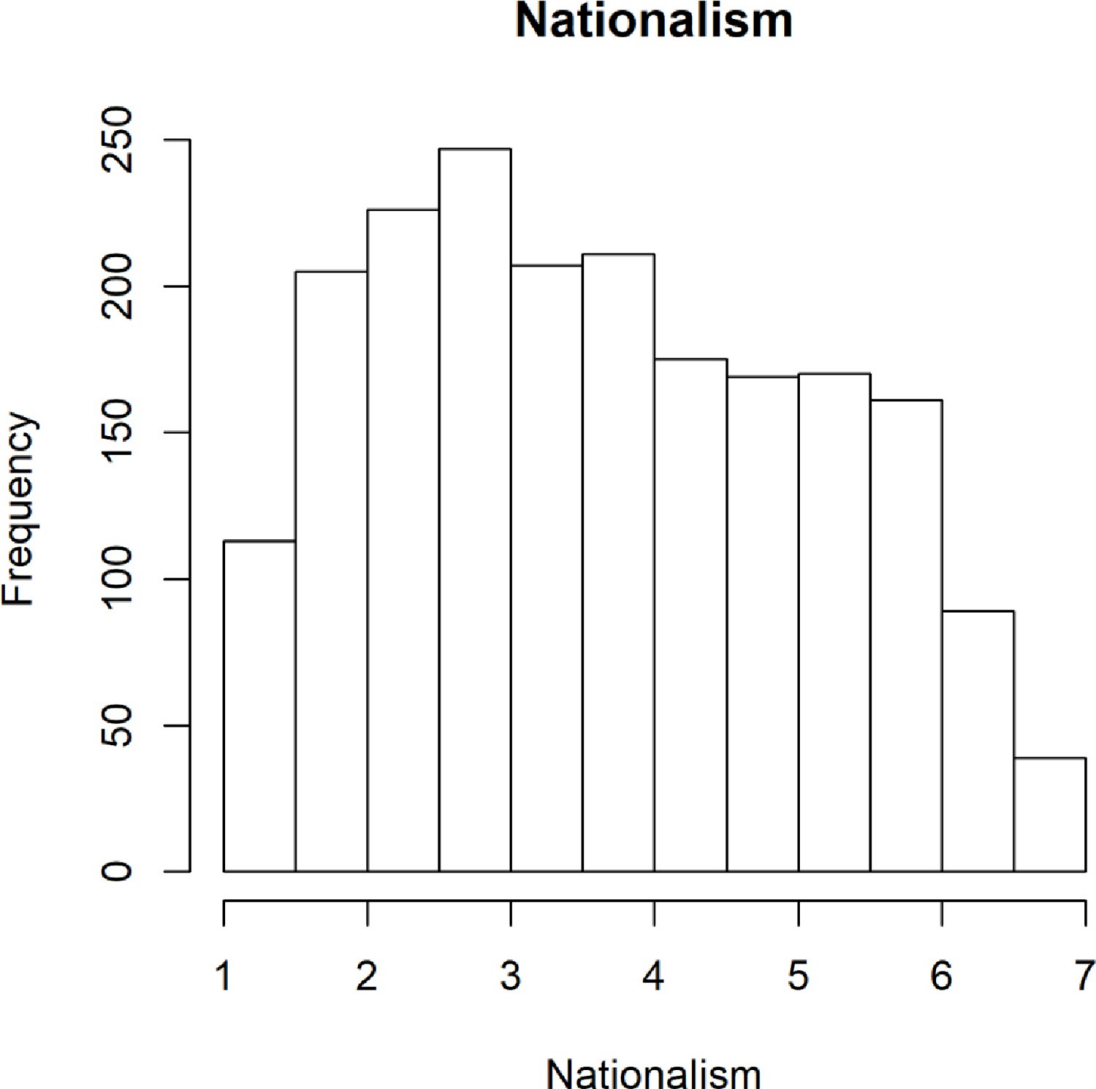
Distribution of nationalist attitudes.

We captured a wide distribution of responses to the nationalism scale detailed below in Fig 6. While the distribution appears normal, it is also possible that because of the social unrest surrounding COVID-19, we are seeing a muted distribution where national identity might be suppressed or re-evaluated, causing there to be more individuals on the extremes of the distribution than in the middle, effectively flattening our normal distribution.

**Fig 7:**
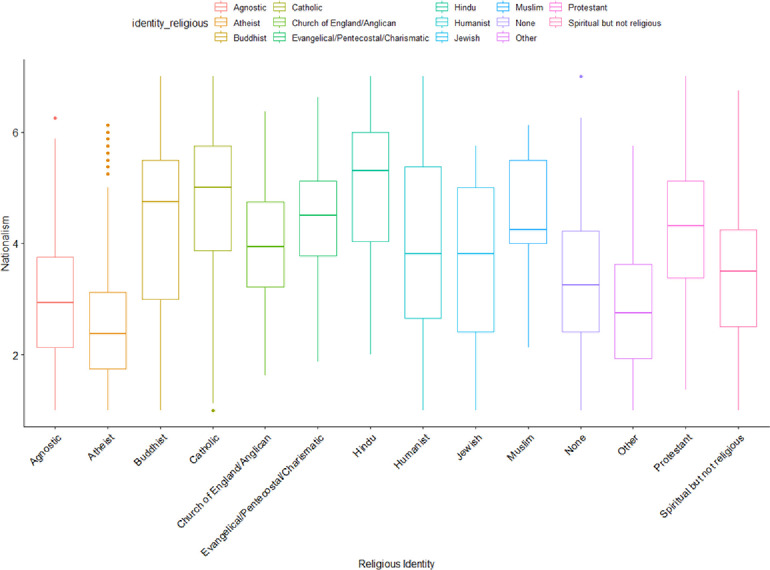
Distributions of nationalism by religious identity.

Generally, we found significant differences between religious groups regarding nationalism (Fig 7). The tendency for some religious groups to be more nationalistic than others indicates that the nonreligious are also more likely to be non-nationalist. This however varies by the group because humanists, for example, are not seen to generally align with Protestants.

**Fig 8:**
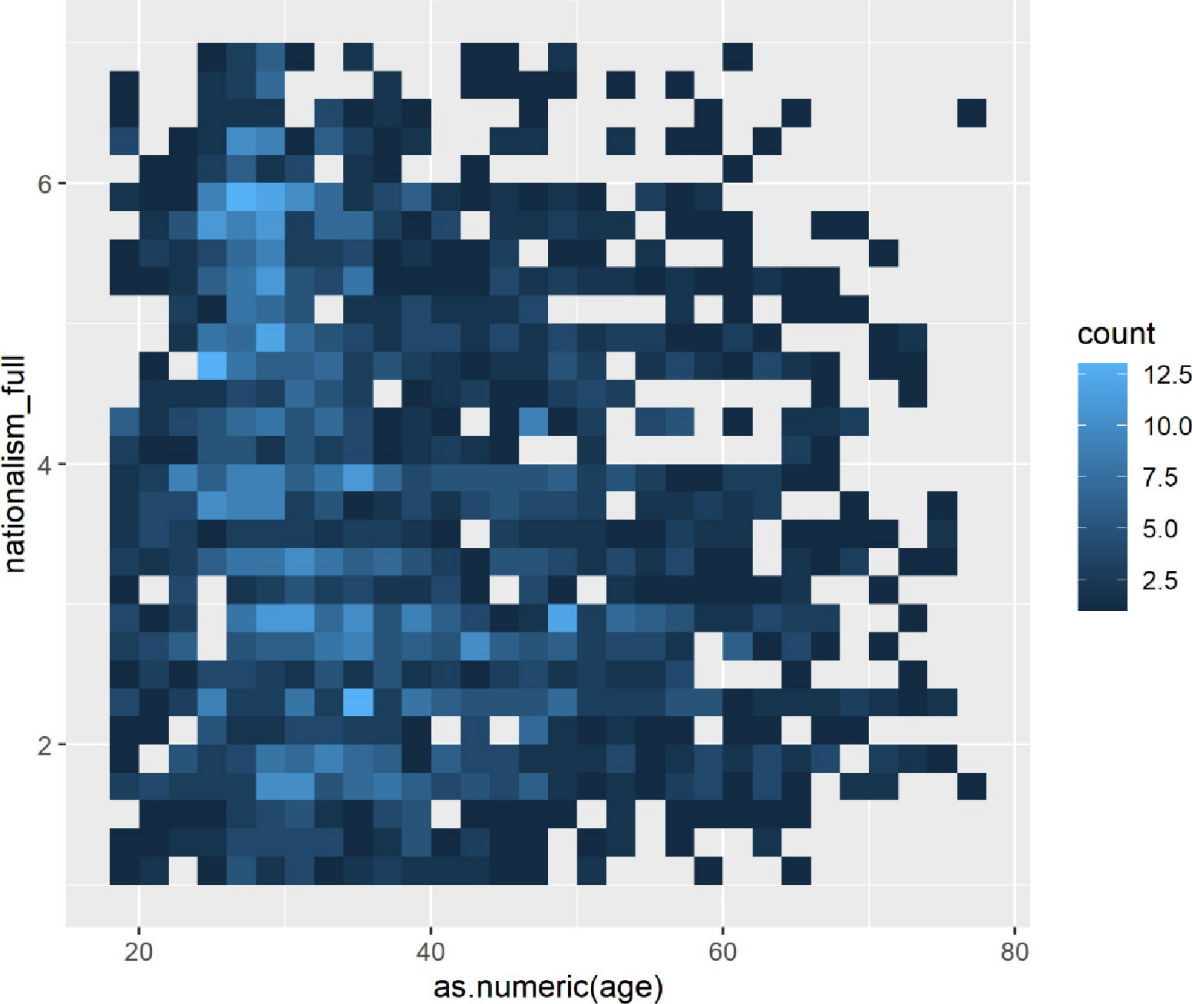
Heat map showing clusters of nationalism by age.

Graphing the participants’ nationalism responses by age, we see that nationalism appears to peak among those in their mid-to-late-20s (Fig 8).

**Fig 9:**
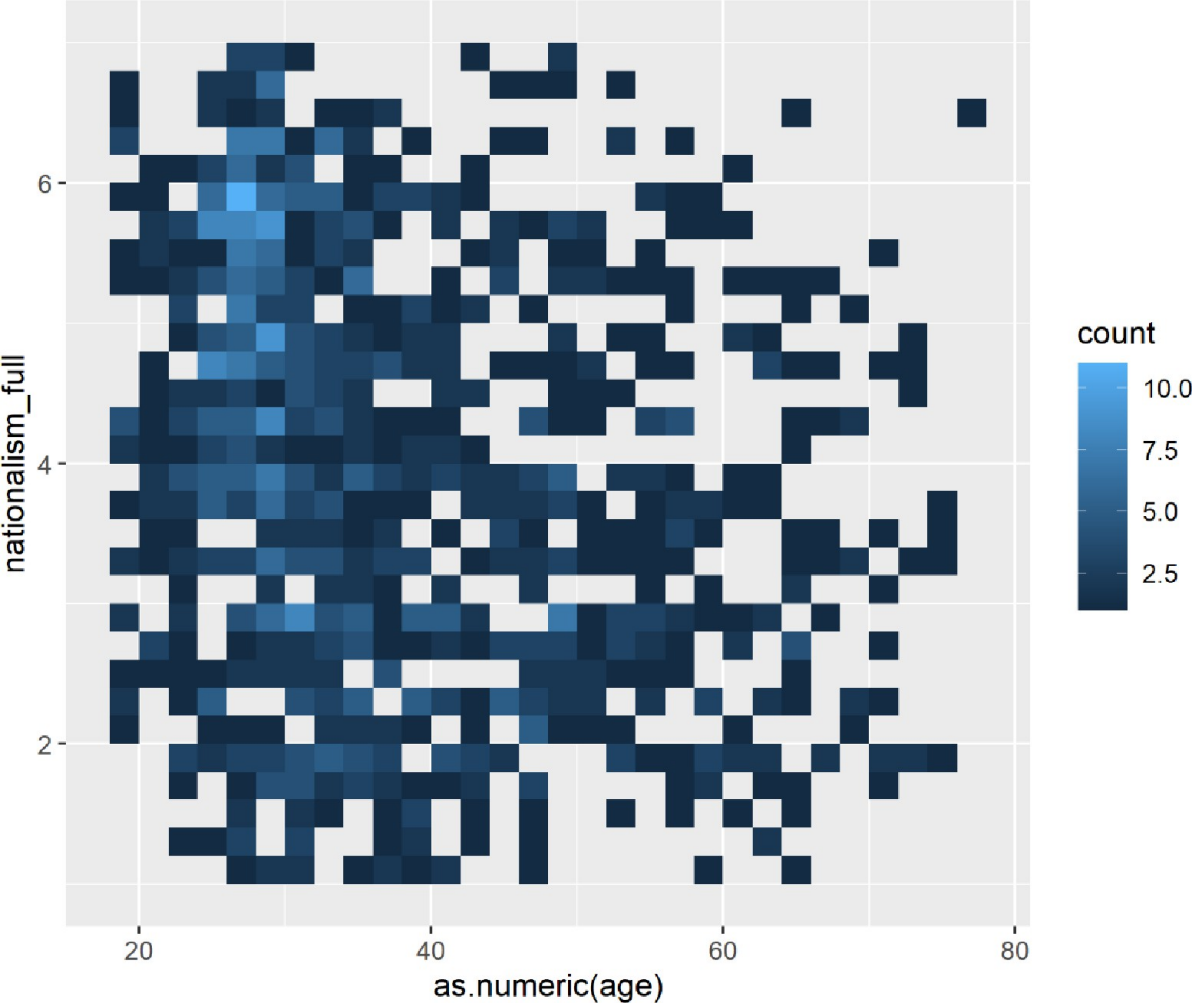
Heatmap of nationalism by age for males.

We further investigated the nationalism measure by looking at men and women separately (Fig 9). For men, we find that the hotspot tends to hold where the greatest nationalist cluster appears at around the age of 25. But nationalism continues well into middle age for men.

**Fig 10:**
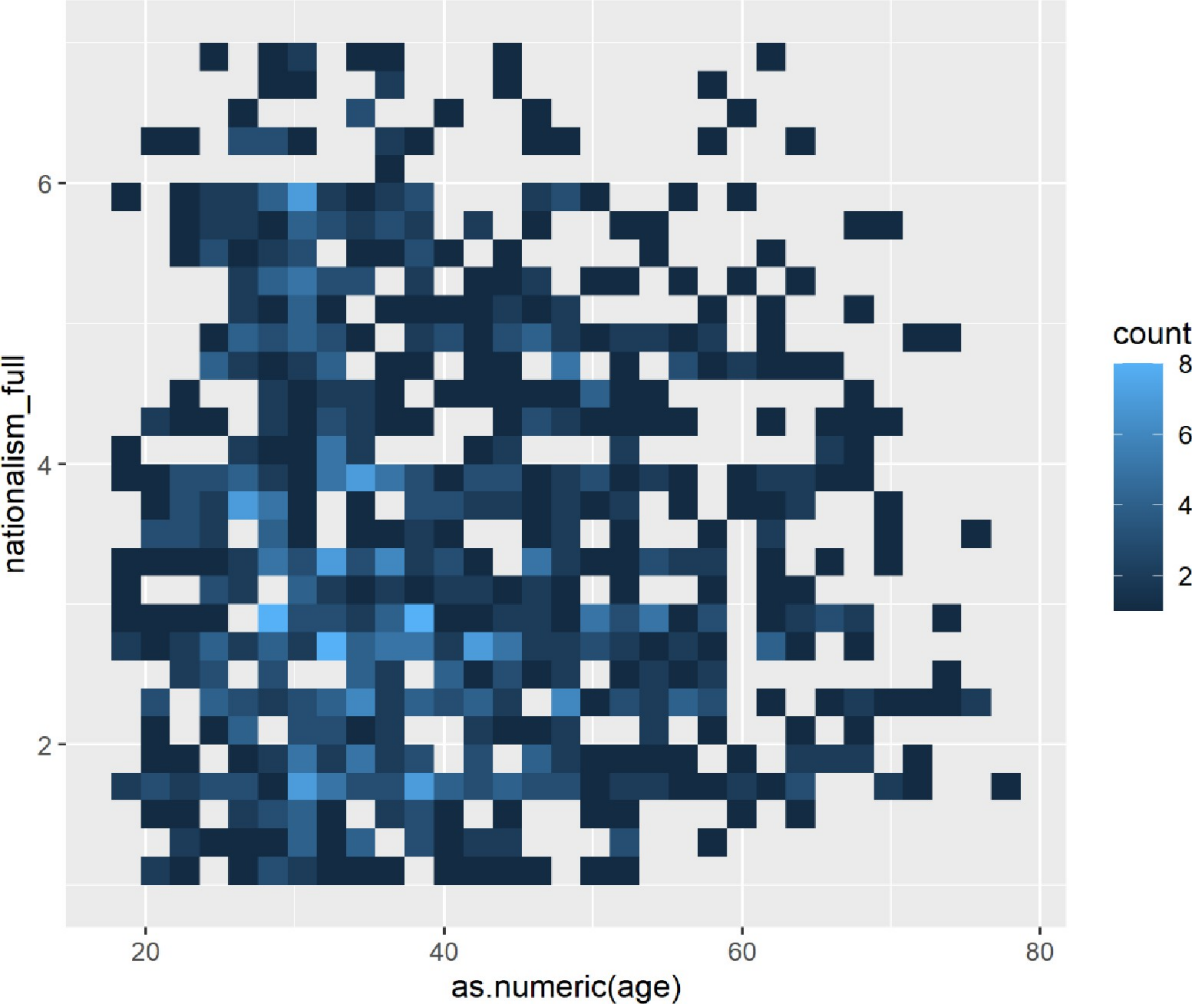
Heatmap of nationalism by age for females.

For women, we see a far less even distribution of nationalism (Fig 10); women tend to cluster lower in the nationalism scale, but the most nationalist women are found around the age of 30. Generally, women’s nationalism clusters in the lower levels of nationalism, with the greatest number appearing below the midpoint, in opposition to the pattern exhibited by men.

Future research is needed to investigate why nationalist tendencies appear differently for each gender and largely correspond to periods of life implicated in major life transitions such as exiting university.

When attempting to predict nationalism as a function of threat, we also found that the different threats had varying effects on nationalism and that controlling for the threat that they believed COVID-19 posed to their way of life increased the model’s predictability significantly (p < .01). Results of these regressions are presented in Table 2.

**Table 2:** Regressions predicting nationalism levels from threats.

Nationalism and Threat		
	Dependent Variable	
	Nationalism Full	
Model	1	2
Threat Predation	0.197***	0.194***
Threat Contagion	-0.417***	0.398***
Threat Financial	-0.210***	0.219***
Threat Natural	-0.310***	0.379***
Threat Social	-0.572***	0.518***
Way of Life Threatened by Covid	--	0.290***
Constant	4.927***	4.200***
Observations	2,000	2,000
R2	0.245	0.287
Adjusted R2	2.44	0.285
Res. Std. Error	1.302 (df = 1994)	1.266 (df = 1993)
F	129.730*** (df = 5; 1,994)	133.875*** (df = 6; 1,993)

Note: * p < 0.1

** p < 0.05

*** P < 0.01.

**Fig 11:**
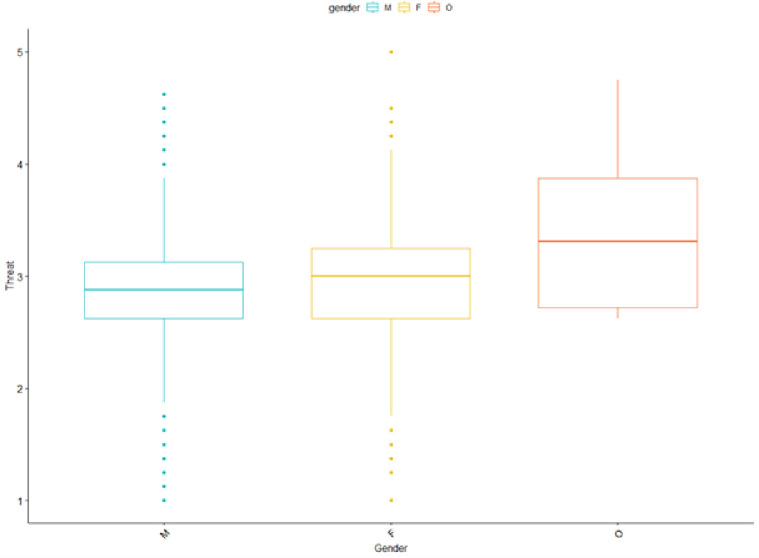
Levels of perceived threat by gender.

The analysis also uncovered a significant difference in perceived threat by gender (Fig 11).

An ANOVA revealed that there was a significant difference between groups (*F*_*(2*,*2002)*_ = 10.25, *p* < .01)) while the posthoc test revealed that the effect was driven by all genders having significant differences (in all pairwise comparisons in a Tukey test *p* < .01).

When analysing the difference in threat perception by nationality, we did find that there was a significant difference between nationalities (*F*_*(10*,*1994)*_ = 1.84, *p* = .05)). Still, a posthoc analysis revealed that the finding is driven exclusively by the difference between Spain and Canada. As such, we are inclined to reject the findings of that model as spurious and continue analyses as pooled given the small sample sizes drawn from those countries and their lesser relevance to the overall research question. We found no significant differences in threat perception by political party support (*p* = .56).

Furthermore, a General Linear Model (GLM) was used to assess the extent to which participant responses were affected by threat perceptions. Table 3 shows our finding that contagion and financial threats have a significant negative relationship with positive attitudes toward immigrants, while social threats have a positive relationship (reminder: the immigration question was binary 0 = no issue; 1 = would not want immigrant neighbours). This suggests that those who perceive more social threats are less likely to want immigrant neighbours, but those who perceive more contagion or financial threats are not.

**Table 3:** Results of GLM to assess effects of threat on anti-immigrant sentiment.

Immigrant Sentiment and Threats		
	Dependent Variable:	
	WVS Immigrants as Undesirable Neighbors	
Threat Predation	0.040***	
Threat Contagion	-0.087***	
Threat Financial	-0.072***	
Threat Natural	-0.038***	
Threat Social	0.113***	
Constant	0.620***	
Observations	2,002	
Log Likelihood	-3,084.61	
AIC	6,181.21	

Note: * p < 0.1

** p < 0.05

*** P < 0.01.

In addition, Table 4 shows that threats have significantly different kinds of effects on different supernatural beliefs. Although all threats were found to be significant, predation and social threats were found to have a positive impact on supernatural beliefs. In contrast, contagion, financial, and natural threats were found to have a negative effect (results for both the original SBS and our extended SBS, where we added items for karma and universal force, are found below).

**Table 4:** Effect of threats on supernatural beliefs.

Supernatural Beliefs and Threat		
	Dependent Variable	
	Supernatural Belief Scale (Origional)	Supernatural Belief Scale (Extended)
Model	1	2
Threat Predation	0.243***	0.194***
Threat Contagion	-0.638***	0.398***
Threat Financial	-0.142***	0.219***
Threat Natural	-0.299***	0.379***
Threat Social	-0.488***	0.518***
Constant	5.433***	4.200***
Observations	2,002	2,000
R2	0.174	0.287
Adjusted R2	0.172	0.285
Res. Std. Error	1.787 (df = 1996)	1.787 (df = 1996)
F (df = 5; 1,996)	84.269***	84.269***

Note: * p < 0.1

** p < 0.05

*** P < 0.01.

Lastly, Table 5 presents the extent to which threats predicted the strength of different religious and national identification styles (namely, identification and fusion). Thus, for identification (with both religious and national affiliations), contagion and financial threats negatively affected identification, while social threats had a positive effect. Natural threats had a negative effect on national identification, but not religious identification; although, this effect was extremely weak. All the threats significantly predicted fusion, with predation and social threats having a positive effect on fusion with both religion and national groups, while contagion, financial, and natural threats had negative effects.

**Table 5:** Predicting levels of religious and national social identity (sid) and identity fusion (ift) as functions of social threats.

Religious and National Identification and Threat				
	Dependent Variable			
	Social Identification with Religion	Identity Fusion with Religion	Social Identification with Nation	Identity Fusion with Nation
Model	1	2	3	4
Threat Predation	0.037	0.222***	0.010	0.168***
Threat Contagion	-0.076*	-0.359***	-0.393***	-0.505***
Threat Financial	-0.080**	-0.179***	-0.151***	-0.197***
Threat Natural	0.040	-0.149***	-0.070*	-0.197***
Threat Social	0.194***	0.438***	0.144***	0.347***
Constant	4.390***	4.414***	6.628***	5.923***
Observations	2,000	1,997	1,995	1,988
R2	0.011	0.11	0.082	0.149
Adjusted R2	0.008	0.108	0.08	0.147
Res. Std. Error	1.659 (df = 1994)	1.624 (df = 1991)	1.406 (df = 1989)	1.503 (df = 1982)
F	4.286*** (df = 5; 1,994)	49.365*** (df = 5; 1,991)	35.719***(df = 5; 1,989)	69.380***(df = 5; 1,982)

Note

* p < 0.1

** p < 0.05

*** P < 0.01.

The role of threats on different personality traits is detailed below in Table 6 using the regressions for each personality trait and each threat.

**Table 6:** Regressions showing the effects of threats on different personality traits from the Big-5 personality measures.

Big-5 Personality Variables and Threat					
	Dependent Variable				
	Openness	Conscientiousness	Extraversion	Agreeableness	Neuroticism
Model	1	2	3	4	5
Threat Predation	-0.026	-0.064***	0.003	-0.071***	-0.151***
Threat Contagion	0.059***	-0.087***	-0.037	-0.171***	-0.163***
Threat Financial	-0.064***	-0.044**	-0.087***	-0.114***	-0.098***
Threat Natural	0.145***	0.081***	-0.017	0.051**	0.126***
Threat Social	-0.197***	-0.098***	-0.027	-0.167***	-0.226***
Constant	3.177***	4.459***	3.425***	4.601***	4.342***
Observations	1,999	2,000	2,001	2,000	2,000
R2	0.071	0.058	0.012	0.11	0.123
Adjusted R2	0.068	0.056	0.01	0.108	0.121
Res. Std. Error	0.850 (df = 1993)	0.726 (df = 1994)	1.002 (df = 1995)	0.857 (df = 1994)	1.018 (df = 1994)
F	30.319*** (df = 5; 1,993)	24.528*** (df = 5; 1,994)	4.902*** (df = 5; 1,995)	49.488*** (df = 5; 1,994)	56.031*** (df = 5; 1,994)

Note: * p < 0.1

** p < 0.05

*** P < 0.01.

No further analysis was undertaken because all the relationships are relatively weak, and explanatory power is low. Moreover, there are debates as to what causal directions, or even feedback loops, might be proposed theoretically, therefore we believe that this could be a fruitful area for future research.

### Structural equation model

To further assess the data and begin to find structure in the data that could be used for informing a system dynamics model, structural equation modelling was conducted to test several models deemed as theoretically interesting or plausible.

**Fig 12:**
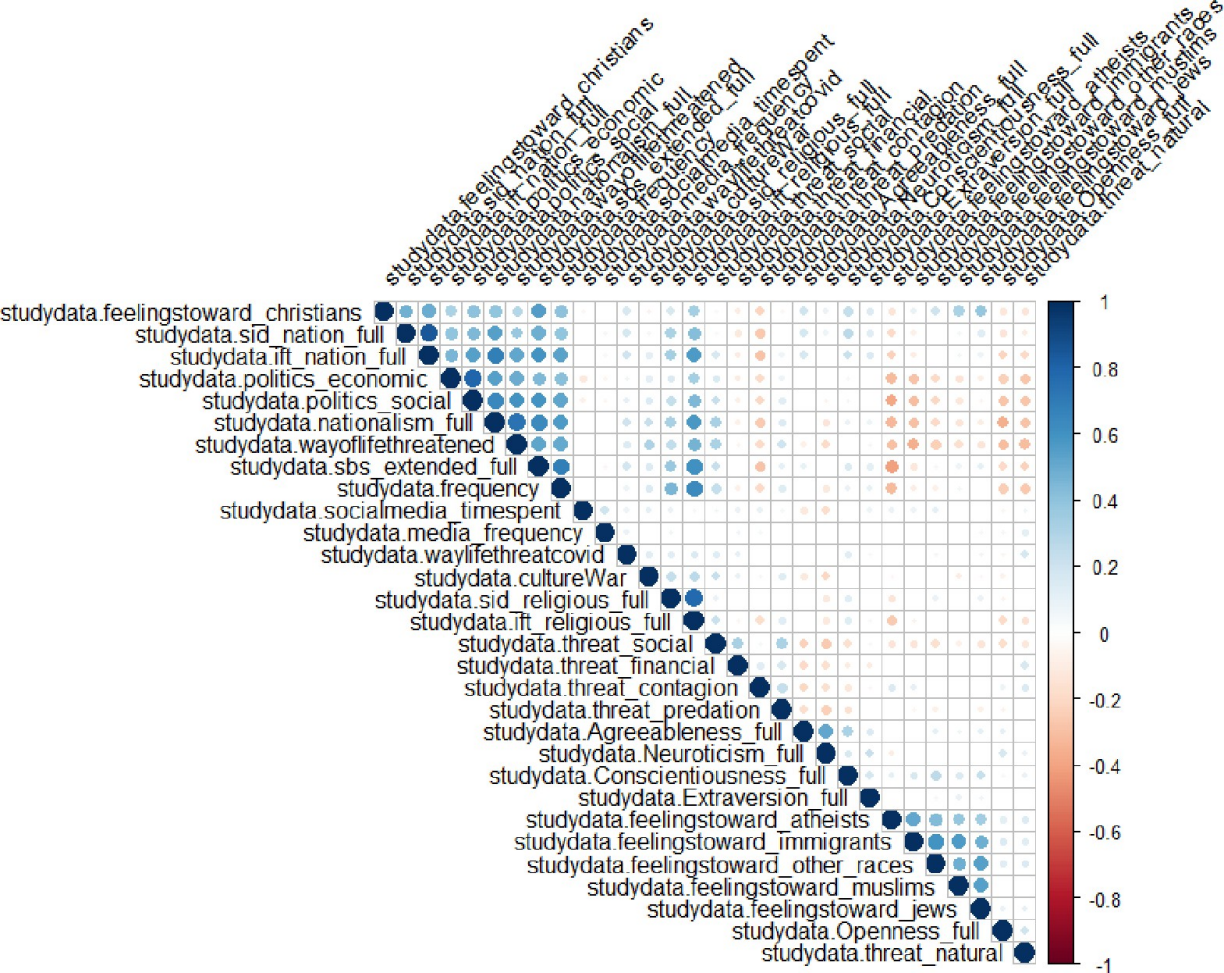
Correlation heatmap of variables in the study used to create our structural equation model.

To create an initial model, given that no theory in the literature has explicitly tackled this issue previously, we utilized exploratory correlations to better understand what variables might be interrelated. This helped us to create the links required between variables in our structural equation model. A full correlation heat map of all the independent correlations found in our dataset is presented in Fig 12.

**Fig 13:**
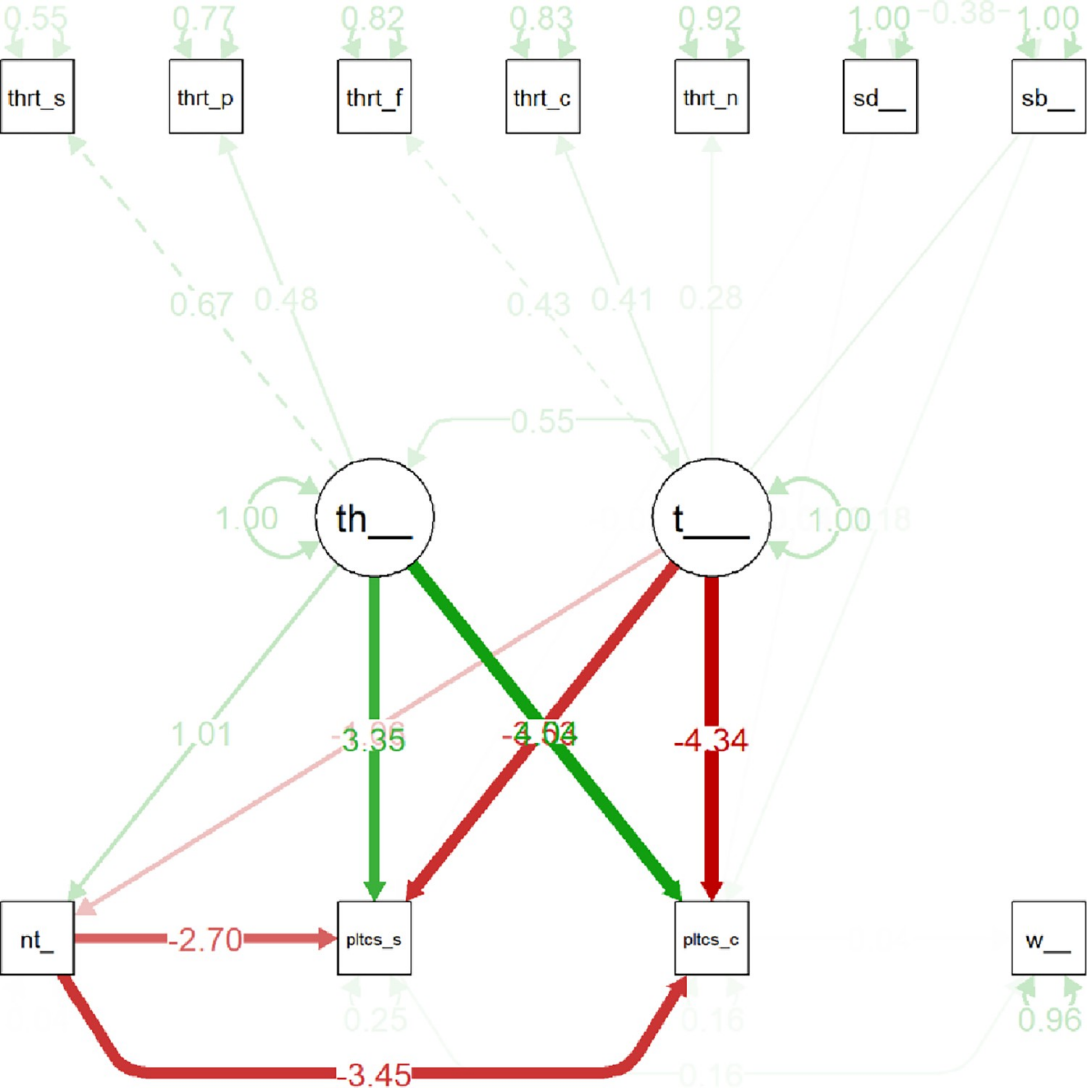
A plausible structural equation model explaining data trends to inform a system dynamics model of nationalism and political sentiment related to threats and supernatural beliefs.

Variations in models were found as an interesting pattern about the threat systems. The analysis, as outlined above, showed that threat does not operate well as a single measure. However, it does seem to function acceptably well as a multi-dimensional scale, with two clusters. In our structural equation modelling (Fig 13), two clusters of threats were shown to have two different and opposing effects on social and economic conservativism. The model that is used to inform a great deal of the system dynamics model is depicted below.

Along the top, there are the 5 threats as well as supernatural beliefs and identity. The two large circles in the middle are latent variables constructed to capture the co-variance in the sub-items of the threat measure. Along the bottom, there is nationalism and social and economic conservativism. On the lower right is the WVS measure for anti-immigrant values. As indicated in Fig 13, social and predation threats form one cluster, which has a positive effect on social and economic conservativism. Meanwhile, financial, contagion, and natural threats have a negative effect, predicting higher levels of liberal political beliefs. These two effects had similar directional effects on nationalism, which also had a negative effect on social conservativism, but a positive effect on anti-immigrant sentiment.

Statistical analysis of the model above (Fig 13) found an acceptable fit (statistical analyses provided in Table 7A and 7B below). This model performed better than most of the other tested models (*AIC* = 55566.78); and it presented itself as the most generalizable theoretical model of those investigated for this pilot study.

**Table 7:** a Factor loadings, regressions, and variances for the Structure Equation Model in Fig 12. b Residuals, latent variances and fit indices for the Structure Equation Model in Fig 12.

	Model			
	Estimate	St. Err	z	p
	**Factor Loadings**			
threat_soc_pred				
threat.social	1.00^+^			
threat.predation	0.81	0.07	12.36	< .01
threat_cont_fin_nat				
threat.financial	1.00^+^			
threat.contagion	0.86	0.11	7.47	< .001
threat.natural	0.56	0.11	5.36	< .001
	**Regression Slopes**			
nationalism_full				
threat.soc.pred	2.75	0.25	11.2	< .001
threat.cont.fin.nat	-3.62	0.26	-13.7	< .001
politics_social				
threat.soc.pred	17.52	32.95	0.53	0.595
threat.cont.fin.nat	-23.23	38.15	-0.61	0.543
nationalism.full	-5.18	10.58	-0.49	0.624
sid.religious.full	-0.04	0.03	-1.21	0.226
sbs.extended.full	0.48	0.04	13.7	< .001
politics_economic				
threat.soc.pred	20.82	28.14	0.74	0.459
threat.cont.fin.nat	-28.2	37.85	-0.75	0.456
nationalism.full	-6.51	9.47	-0.69	0.492
sid.religious.full	-0.08	0.04	-2.13	0.033
sbs.extended.full	0.26	0.04	7	< .001
WVS_undes_immigrants				
politics.social	0.06	0.01	5.01	< .001
politics.economic	0.02	0.01	1.15	0.249
	**Residual Variances**			
threat.social	0.38	0.03	13.74	< .001
threat.predation	0.67	0.03	24.36	< .001
threat.financial	0.87	0.04	23.42	< .001
threat.contagion	0.68	0.03	25.96	< .001
threat.natural	0.71	0.03	28.06	< .001
nationalism.full	0.08	0.04	1.87	0.061
politics.social	2.06	1.98	1.04	0.299
politics.economic	1.29	3.01	0.43	0.668
wvs.undes.immigrants	1.24	0.08	15.9	< .001
sid.religious.full	2.76^+^			
sbs.extended.full	3.87^+^			
+ Fixed parameter				
	Residual Covariances			
sid.religious.full w/sbs.extended.full	1.23^+^			
	Latent Variances			
threat.soc.pred	0.3	0.03	10.43	< .001
threat.cont.fin.nat	0.19	0.04	5.27	< .001
	Latent Covariances			
threat.soc.pred w/threat.cont.fin.nat	0.13	0.03	4.59	< .001
	Fit Indices			
X2	1588.64(34)			< .001
CFI	0.74			
TLI	0.58			
RMSEA	0.15			
+ Fixed parameter				

**Fig 14:**
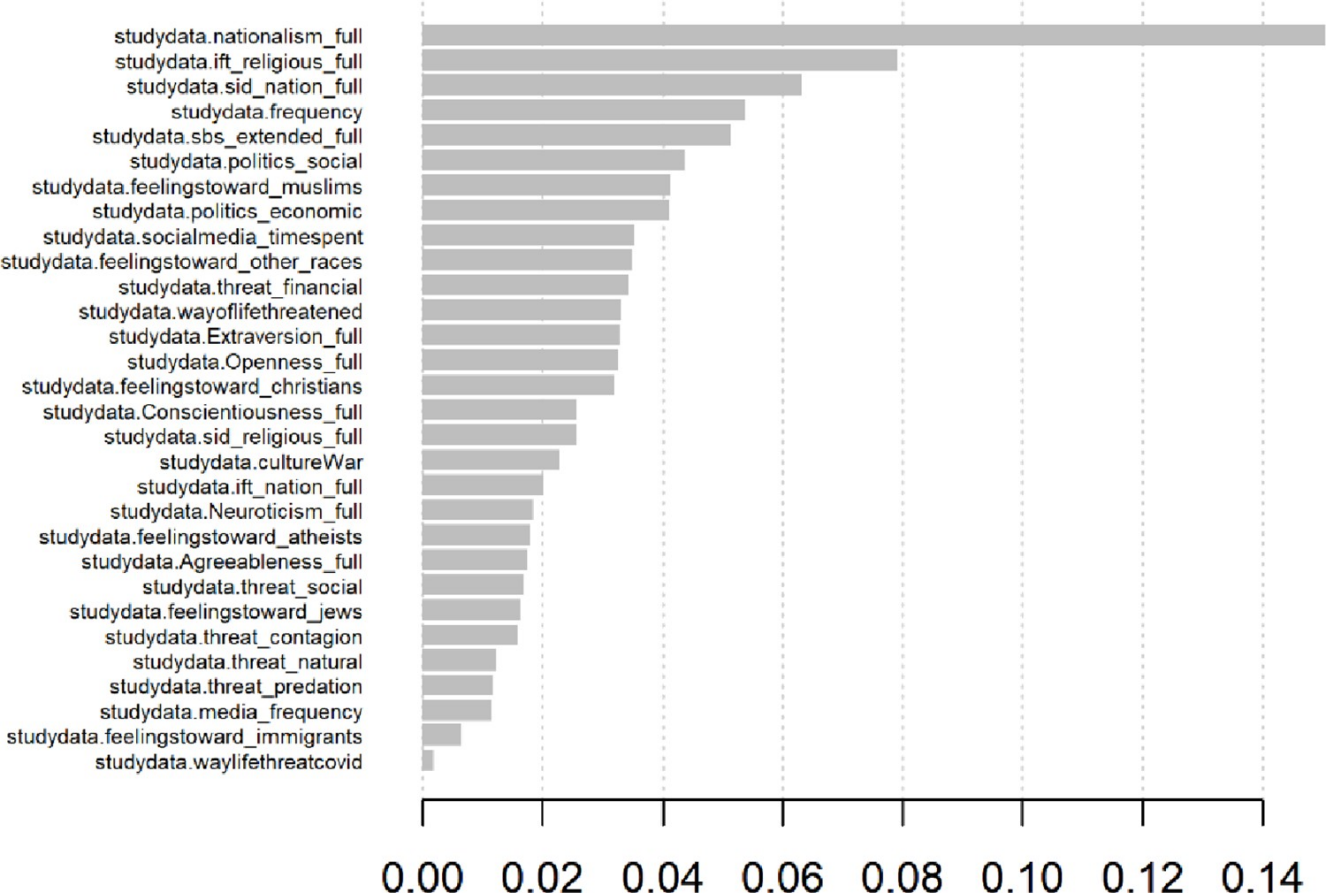
Importance of key factors in predicting if someone will hold anti-immigrant sentiment.

Lastly, we utilized a machine learning system to highlight what features could help us classify participants based on their responses to the WVS immigrant question. In this model (code provided in the electronic appendix) we utilized a machine learning model to fit the data for each participant. The model was fit against the participants acceptance of immigrants as neighbours (a survey question that has been widely deployed in the World Values Survey). We found that the key predictor is nationalism, followed by fusion with one’s religious identity (fusion with one’s national identity is relatively low). This finding is interesting as fusion with religion has been recently studied using AI systems and shows that it can be modelled as an overlapping effect of emotionally laden beliefs about one’s self that are connected with one’s social schema. In particular, it appears to be the effect of long-term reflection on emotional experiences, where one uses the groups beliefs to interpret the significance of the event in their own life. This model suggests that the group boundaries and intense group alignment proposed by identity fusion theory may be manipulated in times of stress to create intense parochial altruism in communities in order to increase feelings of safety. However, the differences between nationalism, and fusion with one’s nation as important factors in this ML system should be the topic of future research. The results are presented in Fig 14.

## Study 2

Study 2 utilized the results of Study 1 to create a system dynamics model that aims to add causal propositions and links to the variables in a way that allows capture of complex feedback dynamics and changes over time, as well as how the system can be affected by psychologically realistic mechanisms, such as habituation to the threats that were so important in the earlier model.

### Methods

The software platform AnyLogic 8 [23] was utilised to construct a system dynamics model. A system dynamics model is a powerful computer simulation technique that is often used to study complex behaviours in multi-variate systems over time. While at its base, a system dynamics model can be reducible to differential and difference equations, this model is presented in a “stock and flow” approach, where the relationships between variables are visually depicted and the directionality (flow) of the model’s causal architecture is more interpretable. The functions were added in raw Java to allow for more accurate calculations and testing. The model itself is a precise way of modelling interactions because it requires that all causal assumptions be formalized (in the computer code itself in addition to the diagrams included here).

**Fig 15:**
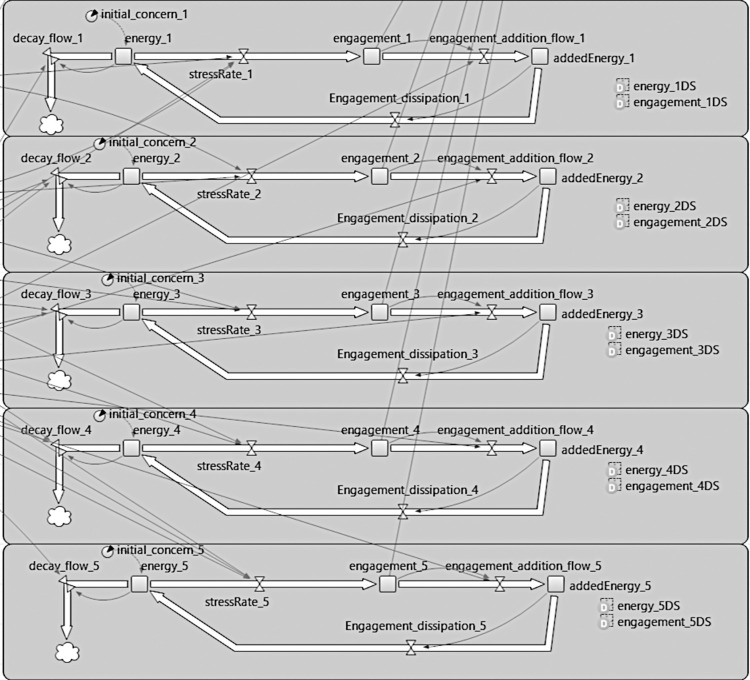
A visual depiction of the system dynamics model used to model threat increases and decreases as functions of stimulus and habituation.

The simulation has two subsystems that work in concert throughout the run of any given simulation. The first system is designed to capture threat perception dynamics. The general form of the model is similar to the one presented and discussed in the simulation of terror management systems in human cognition [2] (and later in [24]) which utilizes a system dynamics model that allows for habituation dynamics to be instantiated. It can also mimic the pattern of the Rescorla & Wagner habituation system. This threat perception system has five subsystems, one for each dimension of threat measured in this study. It is visually depicted in Fig 15 below.

**Fig 16:**
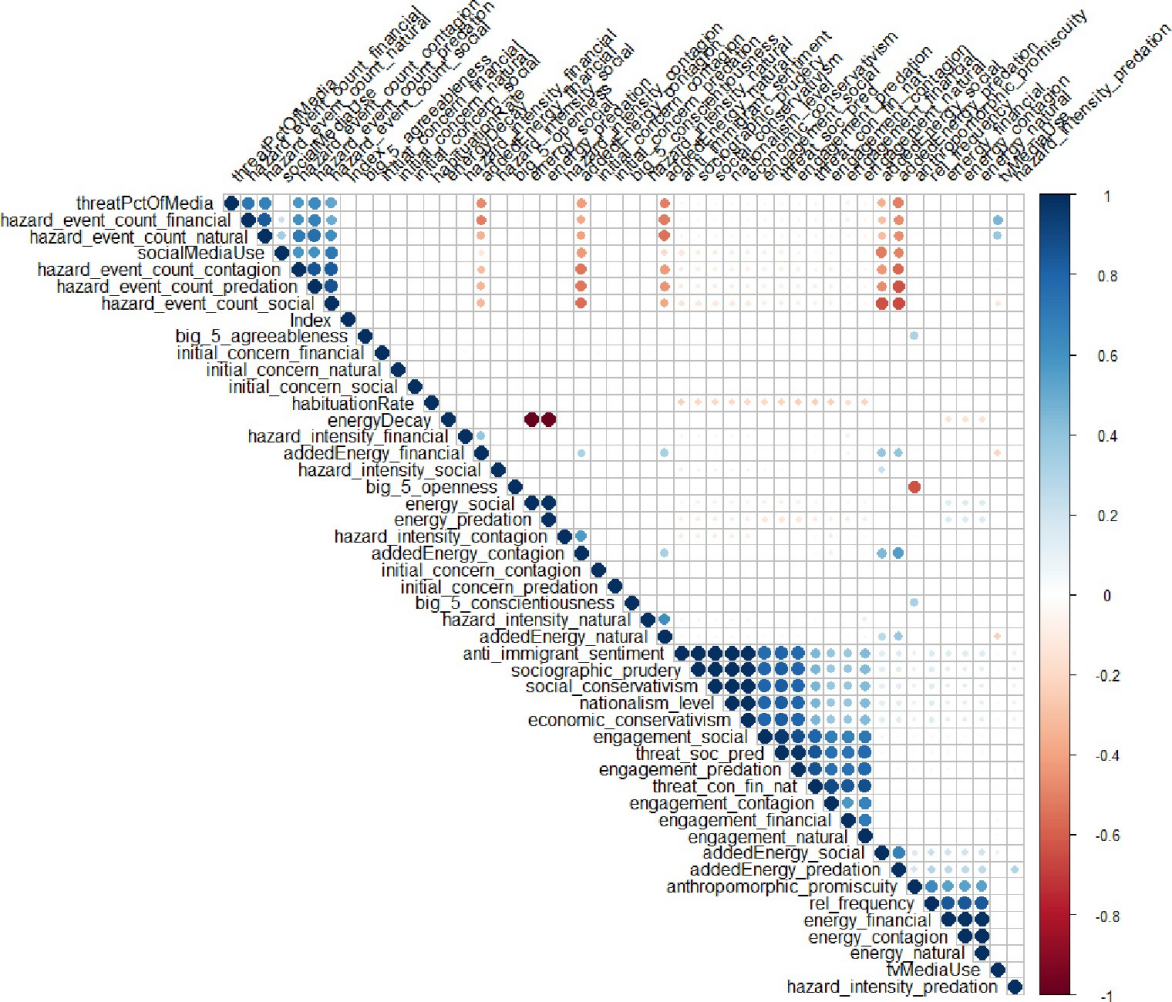
Correlation heatmap of simulation output results.

As the different levels of engagement with threats are calculated, their respective levels are then aggregated into two dynamic variables, mimicking the latent variables in the structural equation model presented in Study 1. These were then posited to interact with the different socio-political variables in the model and psychological tendencies and other individual-level variables and patterns listed in the parameters in the model described below. The structure of the model is depicted in Fig 16 below.

Most of the variables in the figure are self-explanatory, but two require brief clarification (for a more detailed description, see the electronic appendix and source code for the model at https://github.com/alan-analytics/kingstonThreatStudy). As in the article on modelling terror management theory mentioned above [24], “religiosity” was operationalised to designate “socially shared cognitive and ritual engagement with axiologically relevant supernatural agents postulated within one’s in-group.” This sort of imaginative engagement, which promotes cooperation, commitment, and cohesion in the face of out-group threats and environmental challenges, is fostered by two reciprocally reinforcing evolved dispositions: the tendency to infer human-like supernatural causes and the tendency to prefer coalition-favouring moral prescriptions when confronted with ambiguous or frightening phenomena. In other words, religiosity involves the intensification and integration of a hyper-active propensity toward detecting gods as hidden agents and a hyper-active propensity toward protecting in-group norms. We refer to these as “anthropomorphic promiscuity” and “sociographic prudery” respectively (for theoretical background, see [25,26,27,28].

#### Design of experiment

A parameter sweep of the theoretical space of the model was used to test the model. This was achieved by selecting 20,000 uniformly distributed sets of variables for all model parameters (data for each run is included in the electronic appendix). This allowed for statistical tests of the model for all theoretically relevant settings to better understand the causal links proposed in the model and explore the relations among the variables.

Each parameter set was input into the model when a simulation run started. Because of the non-stochastic nature of the system dynamics model, we specified each parameter set only needed to be run once. At the end of each run, data on the input parameters that were used to initialize the simulation, as well as for all outputs related to the number of hazards/threats, nationalism, and political beliefs were saved.

### Results

The data output by the parameter sweep was analysed to better understand the theoretical space of what “could be” using a combination of correlations, regressions, and visualizations. Not all the correlations from the survey are expected to be present in the simulated data because the latter is the result of a specifically defined complex computational model that is reflective of, but not matched by, the complexity of the real world from which the survey data was drawn.

The first analysis that was run was a correlational analysis. The results were then plotted (Fig 16) between all the variables, similar to what was presented for the survey data.

One of the ways that the simulation data can be investigated is through visual representations of relationships under certain partitions. For example, when visualizing immigrant sentiment (x axis) and social conservativism (y axis), but only for simulations where the nationalism was within the lowest quartile, we find that there are instances where extreme anti-immigrant sentiment is possible, but only among individuals who are socially hyper-left-wing. Prima facie, this may be counterintuitive insofar as it challenges common notions of contemporary politics, and the survey data to an extent, but it is consistent with many historical examples; for example, the rise of xenophobia found historically in left wing authoritarian and Marxist states such as in Soviet controlled European nations during the 1900s as well as in China among non-Han Chinese—as evidenced clearly by the genocides of the Tibetans and Uyghurs by the CCP in recent decades. However, in the simulated data, as in history, such extremism is generally rare.

**Fig 17:**
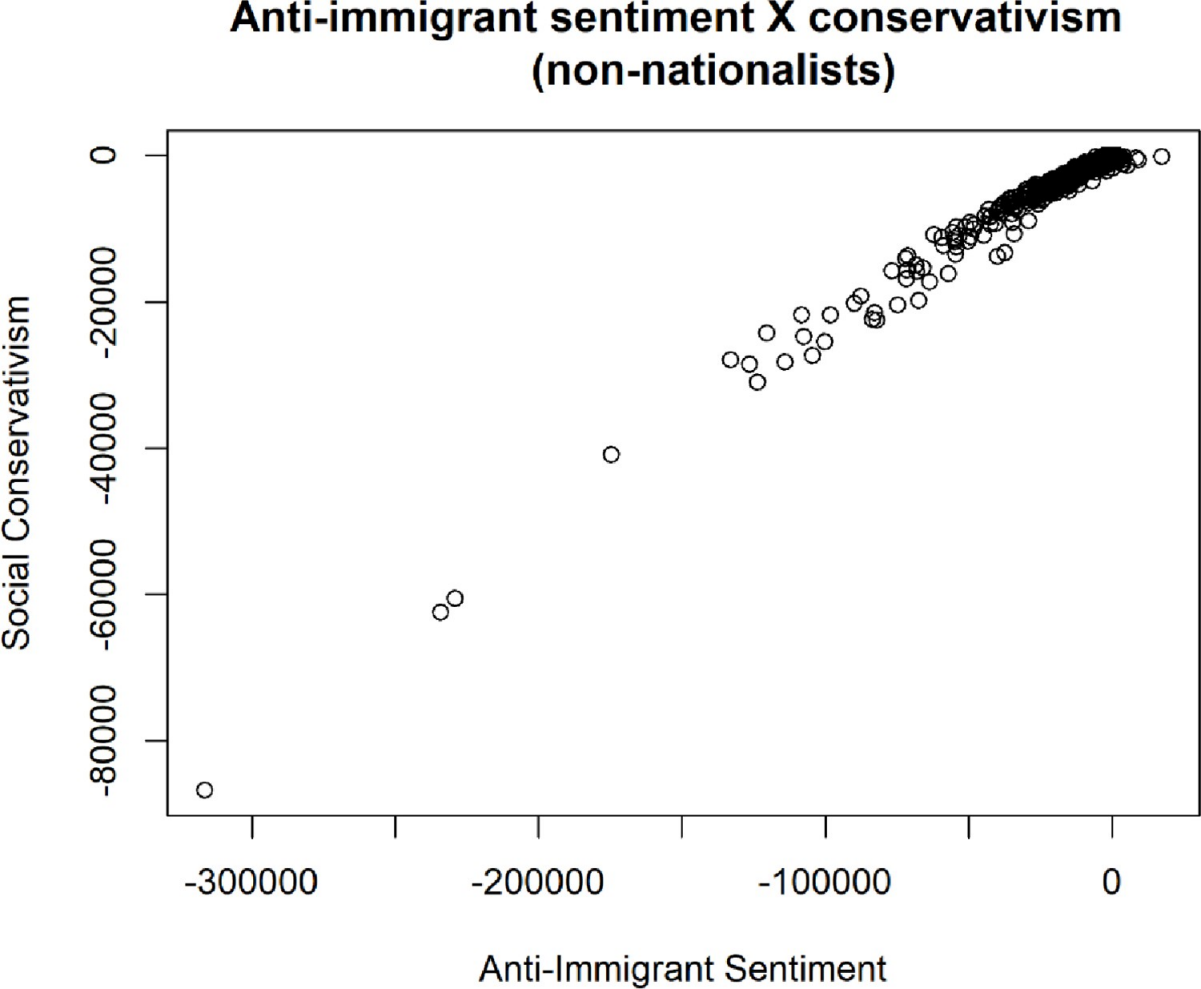
Cluster plot of relationship between liberal (left-wing) social values and anti-immigrant sentiment.

As a point of validation, it was important to find a clustering of causal effects reflected in our simulated data similar to those found in the survey data. Using a regression to test the effects of different threats on anti-immigrant sentiment, the clustering we uncovered is seen below in Table 8 and Fig 17.

**Table 8:** Regression data from simulated data regarding anti-immigrant sentiment as a dependent variable and threat inputs as independent variables.

Simulated Threat and Anti-Immigrant Relationships	
	Dependent Variable:
	Anti-Immigrant Sentiment
Engagement with social threats	694.885***
Engagement with financial threats	-1047.842***
Engagement with contagion threats	-798.107***
Engagement with natural threats	-876.415***
Engagement with predation threats	1,107.319***
Constant	1,864.321***
Observations	20,000
R2	0.846
Adjusted R2	0.846
Residual St. Error	8,891.673 (df = 19,994)
F	21,925.840*** (df = 5; 19,994)

Note: * p < 0.1

** p < 0.05

*** P < 0.01.

**Fig 18:**
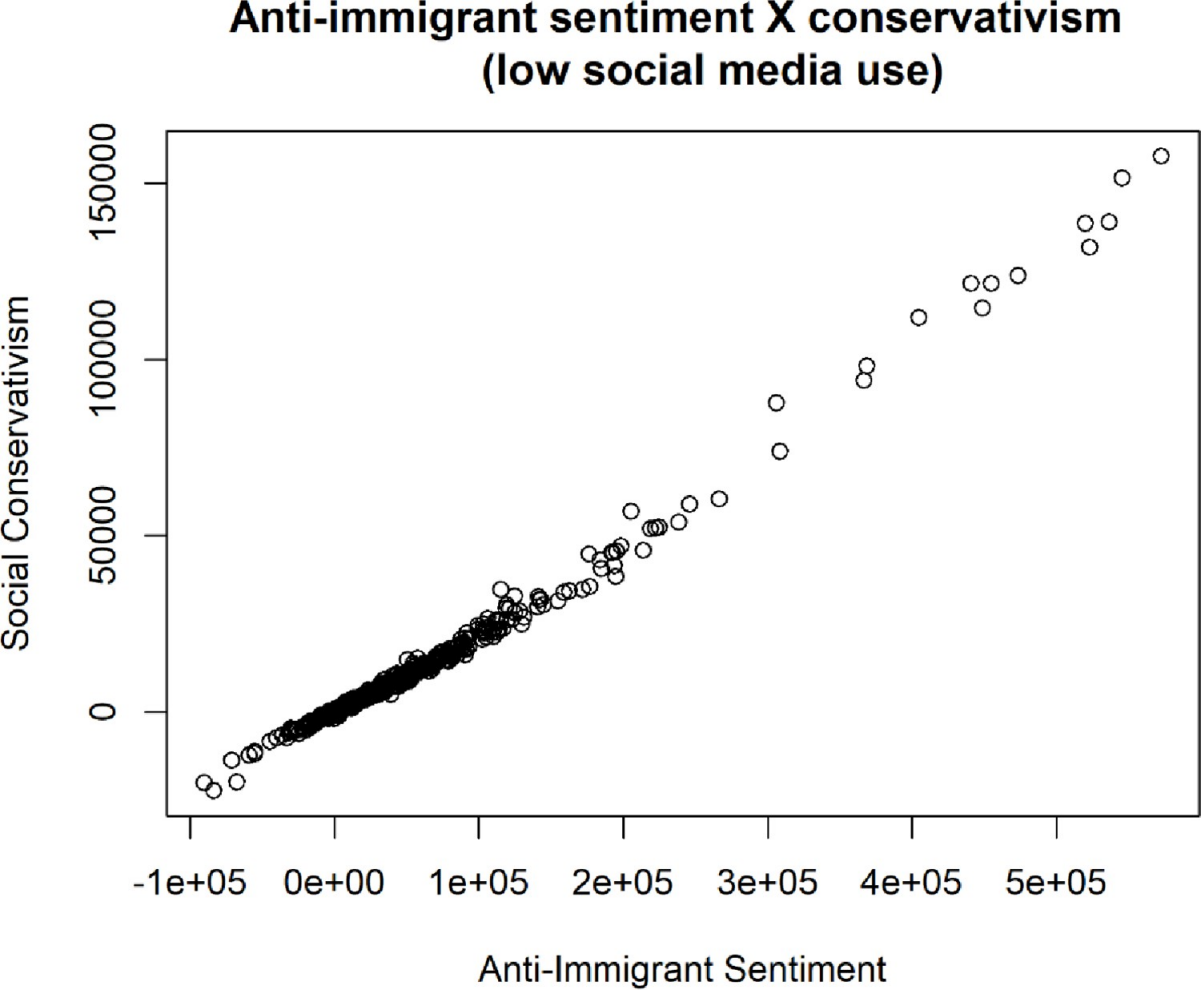
Cluster plot of social conservativism (ring wing social values) and anti-immigrant sentiment.

In addition, further extremes can be found in the relationship between anti-immigrant sentiment and media use detailed below in Fig 18. Among those who are the least frequent consumers of social media, it seems that social conservativism can be extremely high, as can anti-immigrant sentiment.

**Fig 19:**
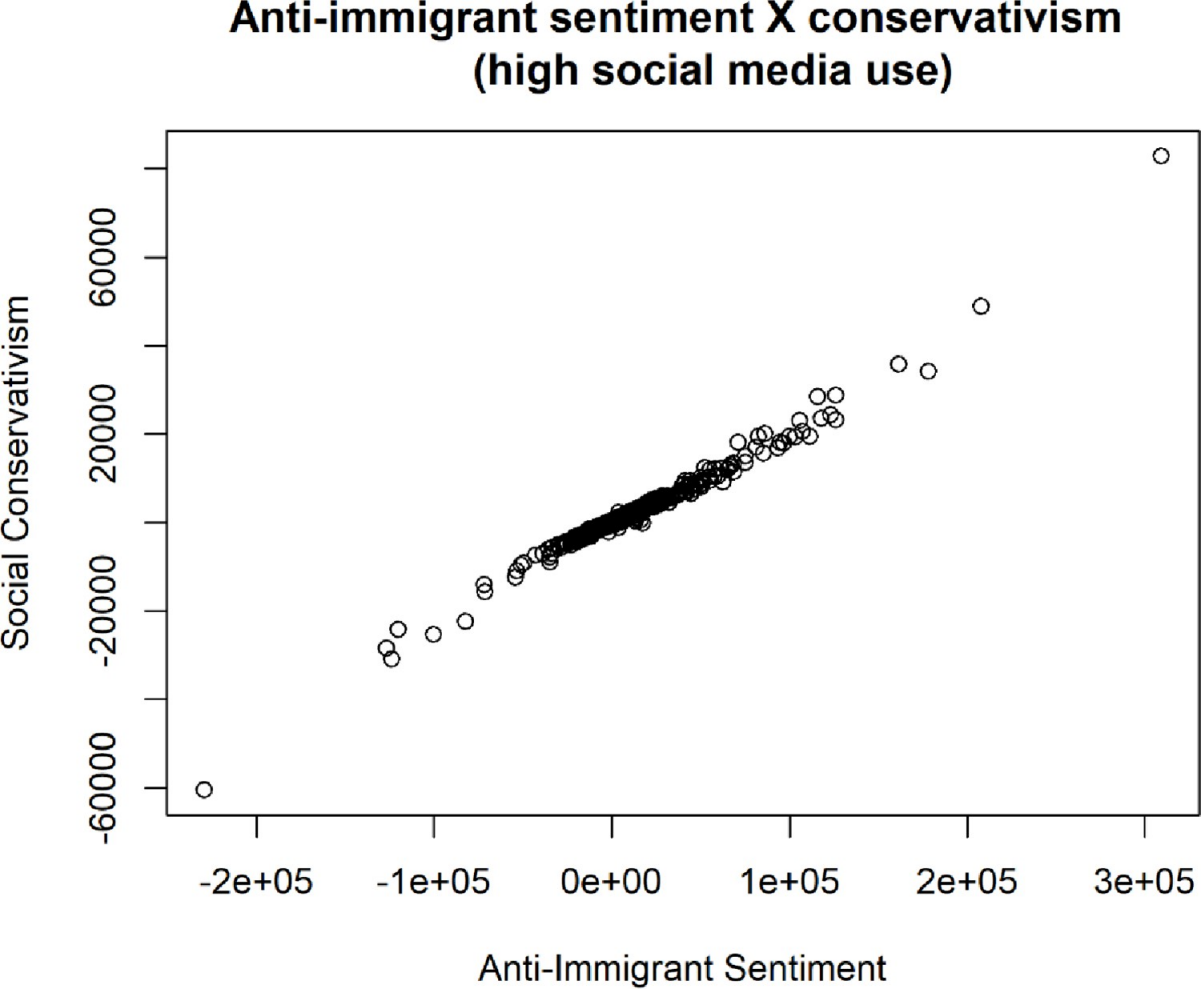
Cluster plot of social political values (both liberalism and conservativism) and anti-immigrant sentiment. On the Y axis, the 0 point could be considered political neutrality, while negative numbers can be considered left-wing and positive numbers can be considered right-wing.

However, among the superusers of social media in the simulated data, Fig 19 how anti-immigrant sentiment can be both positive and negative, depending on the parameters of the simulation. The observed levels of social liberalism (the opposite of social conservativism in the model) are far more extreme than among those who use little social media. This was an unintended outcome but aligns well with earlier surveys and literature suggesting an over-representation of socially liberal ideological frameworks present in online social networks such as Twitter [22].

**Fig 20:**
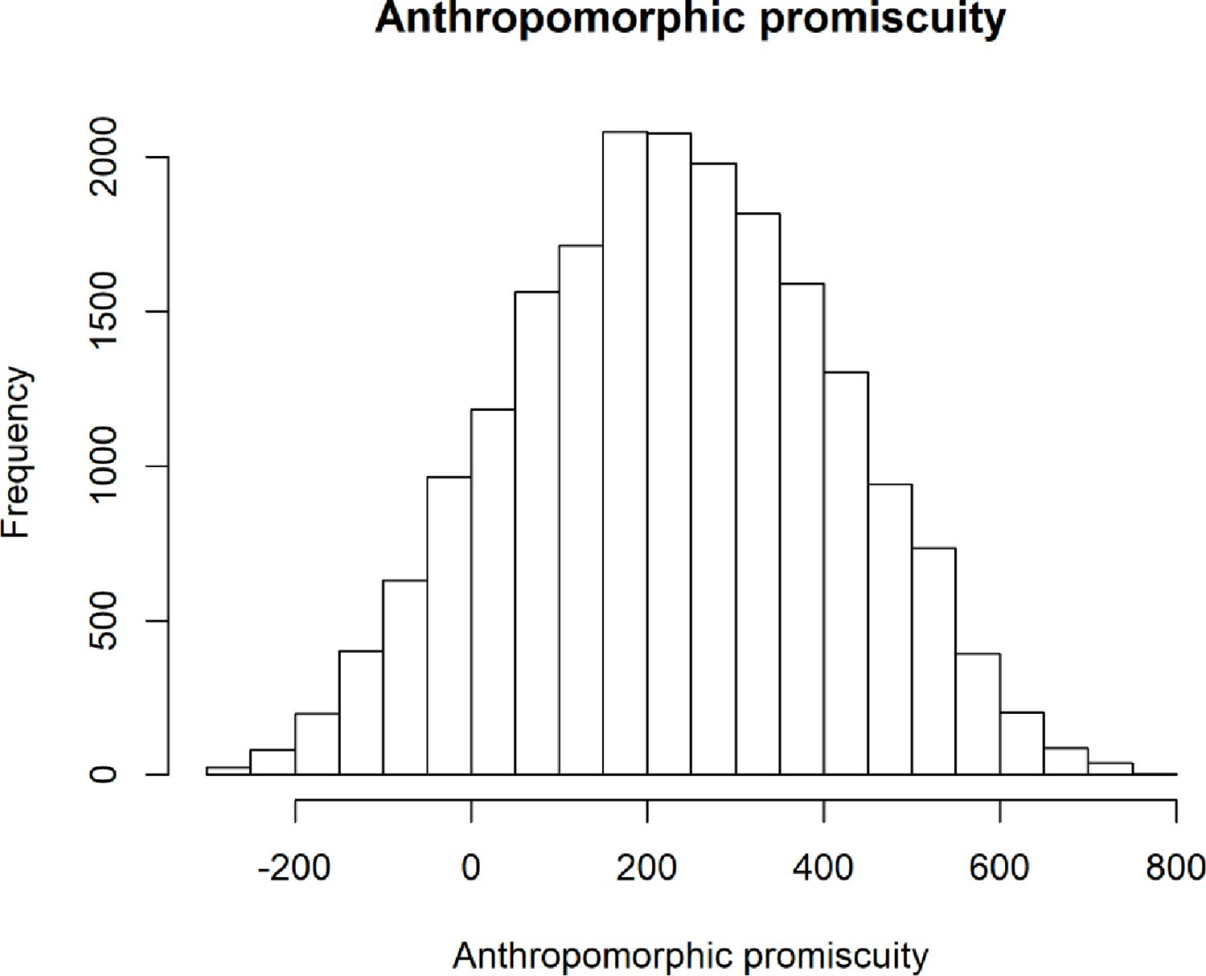
Distribution of anthropomorphic promiscuity outputs from the simulation runs.

Simulated data also uncovered a normal distribution of anthropomorphic promiscuity (Fig 20), with a central tendency toward a relatively low, but positive number. This suggests that given the total theoretical space, most simulations resulted in some god belief, but some simulations exhibited extreme anti-or-pro god beliefs. This seems to reflect real-world patterns as well.

**Fig 21:**
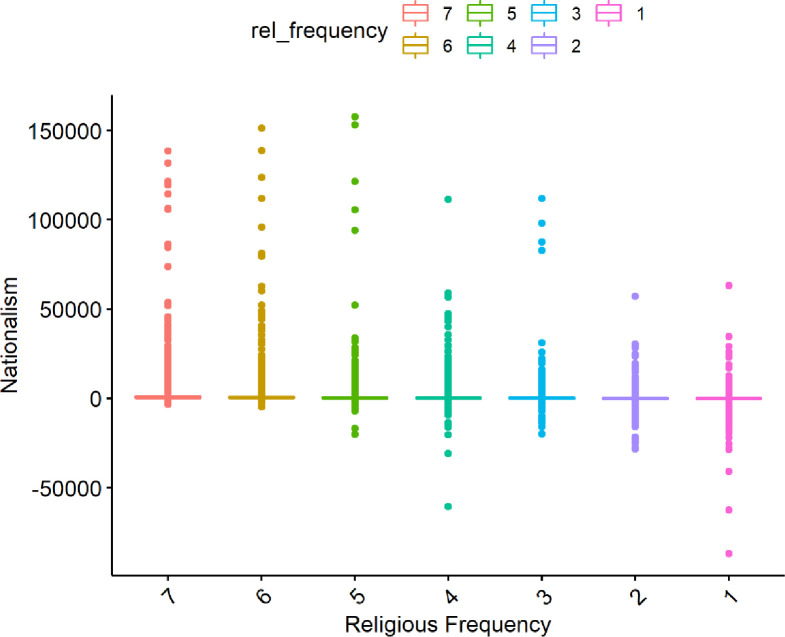
Distribution of religious frequency on nationalism level from the simulated data runs.

Since previous research has found nationalism to be a key indicator of anti-immigrant sentiment, levels of nationalism in the simulated data were also investigated. as well as the relationship between nationalism and religious attendance (visualized below in Fig 21).

Generally, there is a clear trend where more frequent religious attendance can result in higher levels of nationalism. However, in all cases, the levels of nationalism tend to be relatively low. This suggests that, in this simulation, even though there is a positive relationship between religious attendance and nationalism, religious attendance is not a key driver of nationalism, and it has a relatively small effect on the system overall; this appears in line with the real-world data discussed above.

Additional regressions revealed several interesting patterns by sub-setting the data as being either high or low in nationalism. One of the results is that when nationalism is low, there is a significant effect on financial threats on anti-immigrant sentiment, but when nationalism is high there is no significant effect. This prompts the need for more research on the effect of the specific beliefs of a group on its members’ acceptance of immigrants, and how individuals’ framing of their national identity informs the extent to which they’ll accept immigrants.

The results of these regressions can be found in Table 9 below. In the table, model 1 is the entire sample of simulated data, model 2 is only those simulations resulting in low nationalism, and model 3 is only those simulations resulting in high nationalism.

**Table 9:** Regression analysis of anti-immigrant sentiment as it results from other model variables. Model 1 is for all data extracted from the simulation; Model 2 uses data from simulations that only resulted in low levels of nationalism; Model 3 uses only data from those simulations resulting in high levels of nationalism.

Simulated Results Regression for Anti-Immigrant Sentiment				
	Dependent Variable			
	Anti Immigrant Sentiment			
Model	1	2	3	
tvMediaUse	155.293	199.861*	311.24	
threatPctOfMedia	-107.625	-902.908***	462.309	
socialMediaUse	-138.927	-748.730***	-52.424	
rel_frequency	273.552***	291.233***	766.765***	
initial_concern_social	-45.323	69.218	-186.211	
initial_concern_financial	14.363	-91.998	194.417	
initial_concern_contagion	-1,532.591***	-1,859.861***	-2,891.300***	
initial_concern_predation	-15.764	-18.941	-15.237	
hazard_intensity_contagion	-1,532.591***	-1,859.861***	-2,506.320***	
hazard_intensity_financial	-1,407.882***	-1,515.354***	-2,891.300***	
hazard_intensity_natural	-885.580***	-930.353***	-1,638.606***	
hazard_intensity_predation	1,593.59***	1,668.852***	3,235.817***	
hazard_intensity_social	1,162.412***	1,046.521***	2,969.025***	
energyDecay	75.349	12.673	157.79	
big_5_openness	784.327***	820.899***	690.610***	
big_5_conscientiousness	-408.887***	-288.639***	-668.946***	
big_5_agreeableness	-322.506***	-341.067***	-150.31	
hazard_event_count_contagion	-54.260***	-12.87	-204.400***	
hazard_event_count_financial	23.125***	23.008**	31.31	
hazard_event_count_natural	-14.703*	4.00	-80.746***	
hazard_event_count_predation	-53.416***	-7.06	-103.62	
hazard_event_count_social	3.79	-0.46	34.66	
nationalism_level	4.046***	3.985***	3.940***	
economic_conservativism				
social_conservativism				
anthropomorphic_promiscurity				
sociographic_prudery				
Constant	1,672.924***	-219.43	2,530.504***	
Observations	20,000	5,000	5,000	
R2	0.986	0.979	0.989	
Adjusted R2	0.986	0.979	0.989	
Res. Std. Error	2,701.237 (df = 19975)	1,571.367 (df = 4975)	4,338.727 (df = 4975)	
F	57,688.900*** (df = 24; 19,975)	9,728.565*** (df = 24; 4,975)	18,118.520*** (df = 24; 4,975)	

Note: * p < 0.1

** p < 0.05

*** P < 0.01.

Model 1 is for all data extracted from the simulation; Model 2 uses data from simulations that only resulted in low levels of nationalism; Model 3 uses only data from those simulations resulting in high levels of nationalism.

**Fig 22:**
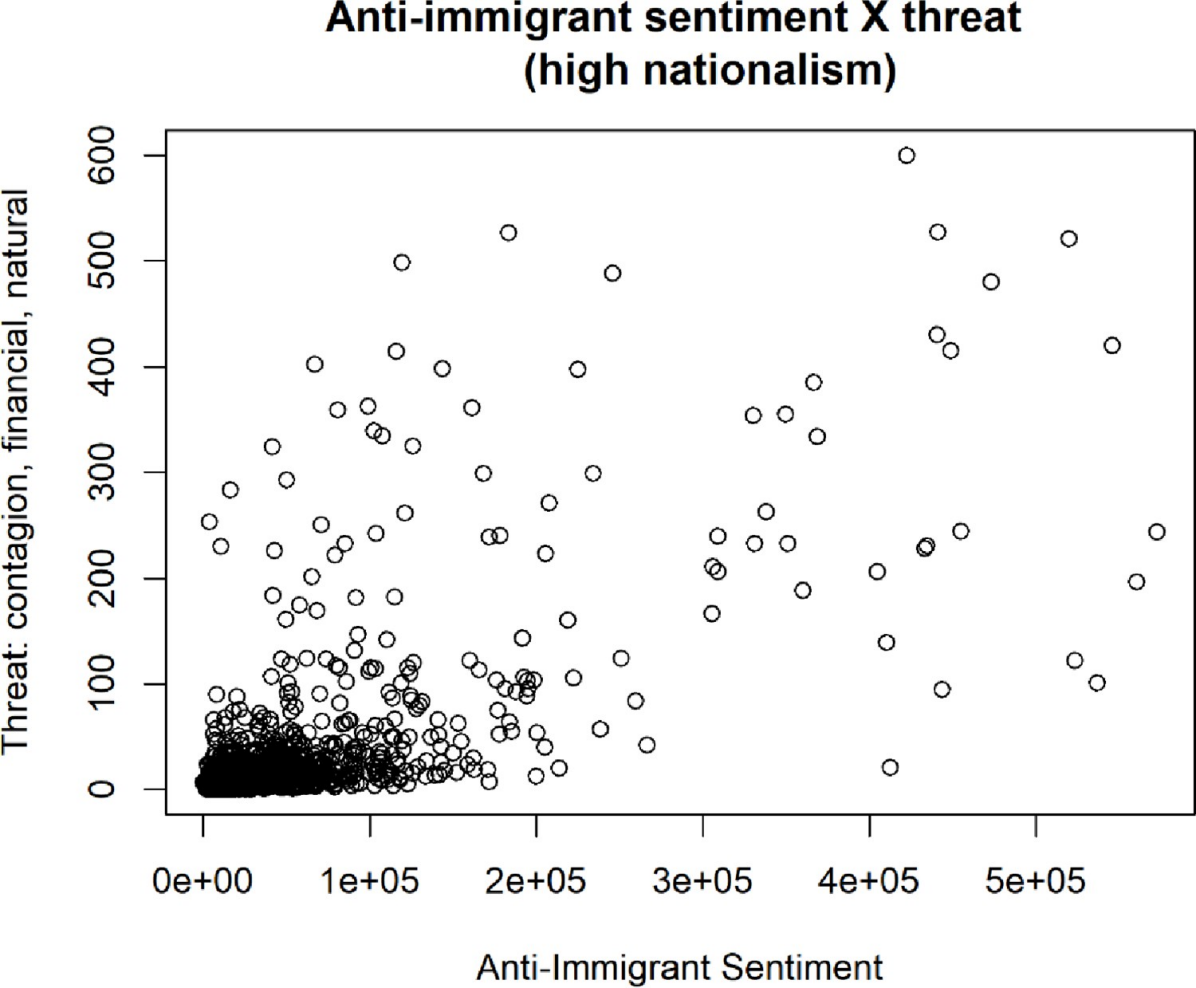
Levels of antiimmigrant sentiment and threat for high nationalism simulations.

**Fig 23:**
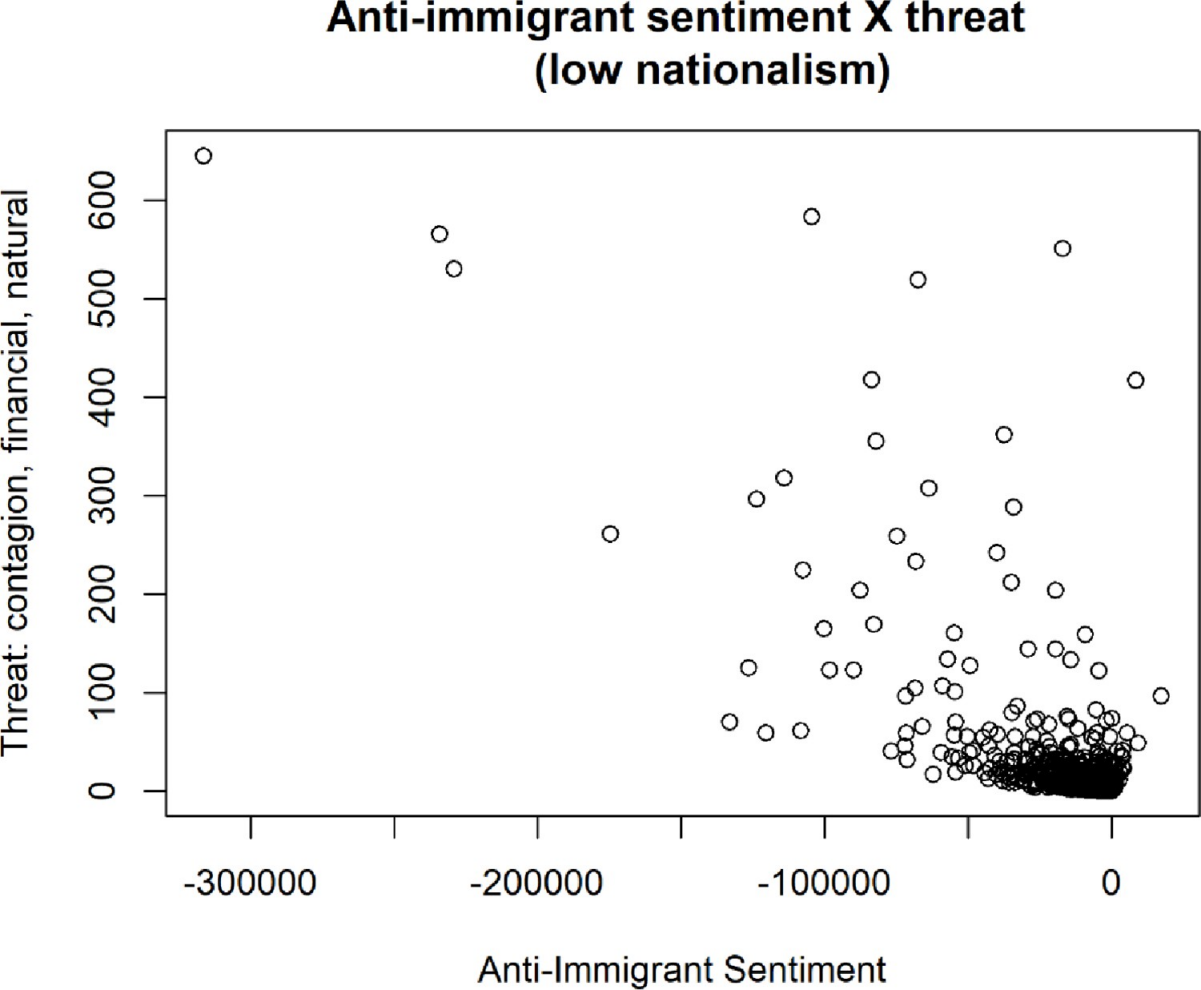
Levels of antiimmigrant sentiment and threat for low nationalism simulations.

To further investigate these relationships, the two nationalism clusters (high Fig 22 and low Fig 23) and their effects on anti-immigrant sentiment as a function of how intense the different threat clusters are were graphed.

What is notable here is that there is a wide range of results, but very clear clusters for the two settings. When visualizing the same dynamics for social and predatory threats, a similar pattern could be observed. In addition, there is a correlation between media use and anti-immigrant values, but it is only significant when religiosity is low. This suggests that religious values are guiding anti-immigrant values in some (but not all) circumstances. The data also seems to suggest that secular ideologies can also have an effect although that effect is different when looking at religious beliefs vs. religious identities. Moreover, those simulations resulting in high anthropomorphic promiscuity (likelihood of attributing events to supernatural agents) are not as affected by the media. But for those who have low anthropomorphic promiscuity, there is a significant negative effect on anti-immigrant sentiment from the percentage of the media that is negative. As such, if we take high and low ending values for sociographic prudery and anthropomorphic promiscuity, we can get 4 separate datasets for further investigation using similar regression techniques as were employed before. In Table 10 below, statistical results for all 4 regressions are presented in full. The data is subset as follows: model 1-low sociographic prudery, model 2-high sociographic prudery, model 3, low anthropomorphic promiscuity, model 4, high anthropomorphic promiscuity.

**Table 10:** Anti-immigrant sentiment as dependent variable in 4 regression models where data is drawn from simulations with low sociographic prudery (model 1), high sociographic prudery (model 2), low anthropomorphic promiscuity (model 3), and high anthropomorphic promiscuity (model 4).

Simulated Results Regression for Anti-Immigrant Sentiment				
	Dependent Variable			
	Anti Immigrant Sentiment			
Model	1	2	3	4
tvMediaUse	199.861*	311.24	248.818	-54.863
threatPctOfMedia	-902.908***	462.309	-468.036**	76.086
socialMediaUse	-748.730***	-52.424	-293.661	-128.884
rel_frequency	291.233***	766.765***	254.587***	295.118***
initial_concern_social	69.218	-186.211	-49.799	-13.979
initial_concern_financial	-91.998	194.417	-7.323	-215.752
initial_concern_contagion	246.201***	364.315*	160.448*	140.938
initial_concern_predation	-18.941	-15.237	108.709	-155.904
hazard_intensity_contagion	-1,859.861***	-2,506.320***	-1,550.441***	-1,540.282***
hazard_intensity_financial	-1,515.354***	-2,891.300***	-1,313.597***	-1,442.491***
hazard_intensity_natural	-930.353***	-1,638.606***	-834.133***	-971.909***
hazard_intensity_predation	1,668.852***	3,235.817***	1,457.795***	1,552.327***
hazard_intensity_social	1,046.521***	2,969.025***	1,064.235***	1,114.912***
habituationRate	1,626.501***	-12,915.230***	-858.049***	-5,332.460***
energyDecay	12.673	157.79	29.151	84.254
big_5_openness	820.899***	690.610***	874.823***	420.12
big_5_conscientiousness	-288.639***	-668.946***	-120.31	-674.821***
big_5_agreeableness	-341.067***	-150.31	-343.592***	-0.67
hazard_event_count_contagion	-12.87	-204.400***	-37.993**	-65.732**
hazard_event_count_financial	23.008**	31.31	23.715**	36.398*
hazard_event_count_natural	4.00	-80.746***	-14.70	-11.33
hazard_event_count_predation	-7.06	-103.62	-17.20	-101.267***
hazard_event_count_social	-0.46	34.66	2.74	-2.27
nationalism_level	3.985***	3.940***	4.126***	3.986***
economic_conservativism				
social_conservativism				
anthropomorphic_promiscurity				
sociographic_prudery				
Constant	-219.43	2,530.504***	448.251*	3,182.368***
Observations	5,000	5,000	5,000	5,000
R2	0.979	0.989	0.982	0.987
Adjusted R2	0.979	0.989	0.982	0.987
Res. Std. Error (df = 4,975)	1,571.37	4,338.73	1,999.83	3,143.57
F (df = 24; 4,975)	9,728.565***	18,118.520***	11,276.280***	15,280.560***

Note: * p < 0.1

** p < 0.05

*** P < 0.01.

In this analysis, it is worth noting that anti-immigrant sentiment is not equally affected by media consumption across all four models. Similarly, while the intensity of perceived threats is significant in all four models, the frequency of perceived events is not, this suggests that some media streams, such as social media, which have been known to promote more incendiary content may increase perceived threat intensity without any issue as to the frequency (which could be a function of the number of online social network contacts also spread that message) as opposed to TV or radio, which would have less frequency, and also have more variability in intensity across situations.

## Discussion

It is important to understand the potential societal effects of the emergence of high levels of anxiety in the wake of the COVID-19 pandemic. In this study, we approached the issue of nationalism—and ultimately social cohesion—by asking how it is affected by various perceived threats in high-stress situations. Since its outbreak in 2019, countries throughout North America, Europe, and Asia have seen widespread unrest; for example, the riots associated with China’s 0-COVID policy in 2022, the Trucker Convoy of Canada, the January 6^th^ protests, and the BLM/Antifa riots in 2020. To understand—and potentially mitigate—further unrest in the future, it is useful to have a better explanation of the underlying causal structures that push and pull the levers of social cohesion, such as identity, religion, nationalism, and out-group hostilities. These larger concerns were the overall motivation for the two studies presented here, which succeeded in elucidating complex issues using new analytical techniques.

The goal of these two studies was to assess the relationships between nationalism, religiosity, and anti-immigrant sentiment during the COVID-19 pandemic from a socio-cognitive perspective. We developed and tested a system dynamics model that theoretically integrated and investigated these relationships, serving as a foundation for future research on the destabilizing effects of contagion threats. The importance of these issues is evident from daily news about the struggle between relatively liberal pan-European institutions and neo-nationalist parties. Many of the latter portrays religion as an important source of their values, which shapes our increasingly socially and politically polarized environment.

Our simulation results suggest that the negative effect of the media and social media on anti-immigrant values is highest when religiosity is low, not when religiosity is high. So, while there is a correlation there between media and anti-immigrant values, it is only significant for people with lower religiosity. This indicates that religious values are guiding anti-immigrant values in some (but not all) circumstances. While no single study is conclusive, this study demonstrates consilience with a variety of similar findings in the psychological and sociological literature discussed above in the introduction. The interdisciplinary nature of this study, combining real-world data collection to create and validate a computational model, also demonstrates that the model itself is not just a mathematical or theoretical object without correspondence to the real world. Rather, the validation of the model shows that the correspondence between the model and data provides a foundation for future studies, such as those involving the world values survey to deploy this model in other contexts, and check for its generalizability beyond the scope of the current study.

Another important finding was that when nationalism is low, there is a significant effect of financial threats on anti-immigrant sentiment, but when nationalism is high there is no significant effect of financial threats on anti-immigrant sentiment. As noted above, this suggests that there is a need for more research on the effect of the specific beliefs of a group on its members’ acceptance of immigrants, and on the way in which individuals’ framing of their national identity informs the extent to which they’ll accept immigrants.

In assessing our threat measures, one of the things that stood out was that, as a scale, our threat measure did not have good validity as a single measure. We found that contagion and financial threats have a significant negative relationship with immigrant attitudes, while social threats have a positive relationship. This suggests that those who perceive more social threats are less likely to want immigrant neighbors, but those who perceive more contagion or financial threats are less likely to respond negatively to questions about having immigrant neighbors. This aligned with a sort of “push and pull” effect regarding how perceived threats affect political beliefs. A similar correlation between COVID-19 threat (presumably a reasonable proxy for contagion threat) and conservativism/liberalism was reported by Su & Shen [4]; however, their categorical approach to political affiliation doesn’t allow for the same statistical comparisons that our multi-dimensional “spectrum” approach utilized, nor does their single threat measure allow for any theoretical interactions or orthogonal effects of other threats (e.g., social, natural, financial).

The survey data leaves several unanswered questions for future research. First, there is a well-documented effect of moral values being correlated with different political persuasions, as discussed briefly in the theoretical background section above. The pattern discovered here between the different threats suggests that evolved human responses to environmental threats and contexts also help to explain social and economic beliefs. Threats may be an underlying cause for the moral domains that are observed in the literature. Future research needs to investigate the relationship between threat and moral domains as causal forces in political ideologies. Ideally, this would be done using controlled or quasi-experimental methods to better untangle the possible causal directions.

The relationships among different styles of identity fusion and nationalism and religiosity should also be investigated. More careful, structured, qualitative research could help to uncover the role of beliefs and semiotically distinct ways of self-identification as potentially important causal factors shaping support for immigration policies. The results of the survey were consistent with earlier literature demonstrating the extreme “groupishness” that exists for fused individuals, as discussed briefly above, but also shed light on previously undocumented effects of media use and content on fusion [29]. Future research can utilize previously deployed methods for analysing fusion as it pertains to ingroup beliefs, which can be inferred from texts and qualitative fieldwork interviews.

The survey data suggested an interesting correlation between religiosity and nationalism. This should be further investigated as both appear to be correlated with anti-immigrant values, although the relationship can be moderated depending on religious group. This indicates that there is a critical role of religious beliefs in that relationship, and the extent to which they are informed by environmental threats is an open question.

The data from the simulation also provide a foundation for additional further research. For example, the simulated data brought to light a very strong negative relationship between openness as a personality trait and anthropomorphic promiscuity. Previous research has shown that some personality variables do correlate with different patterns of belief/non-belief and religious affiliation [30]. However, the extent to which these affect specific dimensions of religiosity, such as anthropomorphic promiscuity and sociographic prudery, is still underexplored.

Both the survey and the simulation suggest that nationalism and religion are affected by the same variables. As such, religion might not be a cause of nationalism (or nationalism the cause of religion), but they could be correlated because of mutual causation. Generally, however, there is a clear trend where more frequent religious attendance can result in higher levels of nationalism. This is probably the most pressing unanswered question of this research. If religion and nationalism are correlated, it could be because they both have some of the same underlying causes. It could be that the same underlying cognitive capacities or tendencies are the foundation for both religion and nationalism, but that religion and nationalism per se have independent effects on anti-immigrant sentiment. If so, it would be problematic to assume that the religious and nationalist effects on anti-immigrant support should be addressed in precisely the same way.

## Supporting information

S1 File https://github.com/alan-analytics/kingstonThreatStudy/blob/master/surveydata_cleaned_FULLAnon. (XLSX)
